# Feruloylacetone and Its Analog Demethoxyferuloylacetone Mitigate Obesity-Related Muscle Atrophy and Insulin Resistance in Mice

**DOI:** 10.1021/acs.jafc.4c07798

**Published:** 2025-01-04

**Authors:** Yen-Chun Koh, Han-Wen Hsu, Pin-Yu Ho, Wei-Sheng Lin, Kai-Yu Hsu, Anju Majeed, Chi-Tang Ho, Min-Hsiung Pan

**Affiliations:** † Institute of Food Sciences and Technology, National Taiwan University, 10617 Taipei, Taiwan; ‡ Department of Food Science, National Quemoy University, 89250 Quemoy, Taiwan; § Sami-Sabinsa Group Limited, Bengaluru 560058, Karnataka, India; ∥ Department of Food Science, Rutgers University, New Brunswick 08901, New Jersey, United States; # Department of Medical Research, China Medical University Hospital, China Medical University, 40402 Taichung, Taiwan

**Keywords:** feruloylacetone, demethoxyferuloylacetone, curcuminoids, muscle atrophy, insulin resistance, gut microbial guild

## Abstract

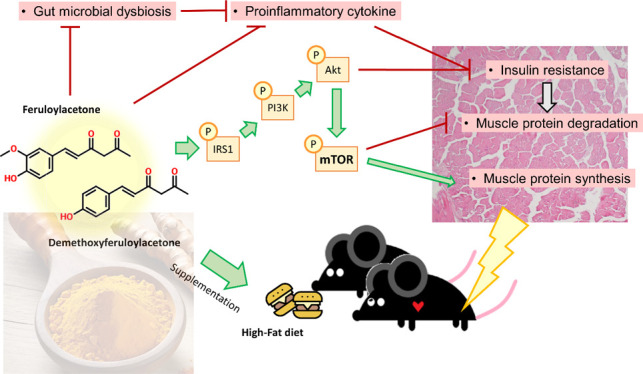

Obesity-induced muscle alterations, such as inflammation, metabolic dysregulation, and myosteatosis, lead to a decline in muscle mass and function, often resulting in sarcopenic obesity. Currently, there are no definitive treatments for sarcopenic obesity beyond lifestyle changes and dietary supplementation. Feruloylacetone (FER), a thermal degradation product of curcumin, and its analog demethoxyferuloylacetone (DFER), derived from the thermal degradation of bisdemethoxycurcumin, have shown potential antiobesity effects in previous studies. This study investigates the impact of FER and DFER on obesity-related glucose intolerance and muscle atrophy. High-fat diet (HFD) feeding resulted in muscle mass reduction and increased intramuscular triglyceride accumulation, both of which were mitigated by FER and DFER supplementation. The supplements activated the PI3K/Akt/mTOR signaling pathway, enhanced muscle protein synthesis, and decreased markers of muscle protein degradation. Additionally, FER and DFER supplementation improved glucose homeostasis in HFD-fed mice. The supplements also promoted the formation of a gut microbial consortium comprising *Blautia intestinalis*, *Dubosiella newyorkensis*, *Faecalicatena fissicatena*, *Waltera intestinalis*, *Clostridium viride*, and *Caproiciproducens galactitolivorans*, which contributed to the reduction of obesity-induced chronic inflammation. These findings suggest, for the first time, that FER and DFER may prevent obesity-related complications, including muscle atrophy and insulin resistance, thereby warranting further research into their long-term efficacy and safety.

## 1 Introduction

Skeletal muscle accounts for around 50% of the total human body mass, and can be considered the largest organ. It plays an essential role in glucose metabolism and is responsible for more than 80% of postprandial glucose uptake, regulating glucose homeostasis via an insulin-dependent mechanism.^1−3^ Both adipocytes and muscle fibers express glucose transporter proteins 1 and 4 (GLUT1 and GLUT4) but the GLUT4 isoform is more abundantly expressed in skeletal muscle. Membrane translocation of GLUT4 is responsible for glucose transportation after insulin-stimulation in muscle fibers.^4^ AMP-activated protein kinase (AMPK) and Calcium/calmodulin-dependent protein kinase II (CaMKII) act as the key kinases to increase the transcription of GLUT4 via hyperacetylation on GLUT4 by Histone deacetylases (HDAC) 4/5,^5^ whereas both the Insulin receptor substrate 1/phosphoinositide 3-kinase/protein kinase B (IRS1/PI3K/Akt) pathway and AMPK activation could promote membrane-translocation of GLUT4.^6^

The modern lifestyle and eating habits have contributed to the “globesity” epidemic and, due to the strong correlation between obesity and type II diabetes (T2DM), weight reduction is an essential strategy for its prevention and management.^7^ It has been reported that severe obesity may be accompanied by impairment of insulin-stimulated skeletal muscle glucose uptake.^8^ Fatty acid overload in nonadipose tissues like skeletal muscle caused by obesity might result in elevation of reactive oxygen species (ROS) and mitochondrial dysfunction, both pathologies of insulin resistance.^9^ In other words, disruption of the balance between fatty acid oxidation and synthesis within skeletal muscle could cause insulin resistance.^10^

Physiological changes associated with obesity include loss of muscle mass and functionality, changes which may accompany inflammatory response and metabolism dysregulation, and insulin insensitivity.^11^ Furthermore, myosteatosis, or ectopic fat deposition in skeletal muscle, is negatively correlated with the mass and strength of skeletal muscle, and can accompany systemic metabolic imbalances like insulin resistance.^12^ In a clinical study, a positive association was found between sarcopenia, a type of muscle loss that occurs with immobility and aging, and increased insulin resistance in obese individuals with higher levels of Homeostatic Model Assessment for Insulin Resistance (HOMA-IR) and Hemoglobin A1C (HbA1C).^13^ More importantly, insulin resistance is more strongly linked to sarcopenic obesity than to sarcopenia or obesity alone in both young and old adults.^13^ Moreover, the prevalence of obesity among children and adolescents has increased dramatically in the modern age.^14^ The term obesity with low lean muscle mass (OLLMM) has been proposed to describe the phenomenon of muscle loss in obese individuals across all age groups.^15^ Enlarged adipocytes during obesity contribute to a low-grade inflammatory state by secreting various adipokines and cytokines. This chronic inflammatory environment plays a crucial role in the development of insulin resistance and the progression of obesity-related sarcopenia.^16^ Loss of function for glucose uptake and utilization promotes obesity and the cytokines secreted accelerates muscle catabolism, in return, muscle mass loss is responsible to decreased insulin-responsive target tissue. Impaired glucose uptake and utilization contribute to obesity, while the cytokines secreted by adipose tissue accelerate muscle catabolism. This muscle loss further reduces the mass of insulin-responsive tissues, exacerbating insulin resistance.^16^

As a subclinical and multidimensional disease, sarcopenic obesity is clinically identified and diagnosed without universal consensus.^17^ There is currently no curative therapeutic strategy for sarcopenic obesity beyond lifestyle improvements such as exercise, diet, and supplementation to enhance muscle composition and function. This is due to the unclear mechanisms underlying the disease onset.^17^ Sarcopenic obesity diminishes quality of life and increases mortality risk due to the challenges associated with its treatment. While some supplements and drugs show promise, current strategies include targeting NF-κB for inactivation, AMPK or glutathione (GSH) for activation, and sphingosine-1-phosphate (S1P) receptors. Additionally, micronutrients and minerals are considered beneficial.^17^ An increase in whole-body proteolysis has been observed in obese women compared to those who are nonobese,^18^ and maintaining or increasing muscle mass could be a potential strategy to prevent sarcopenia.^19^ Mammalian target of rapamycin (mTOR) is recognized as a key regulator to maintain skeletal muscle mass by controlling protein synthesis.^20^ Its upstream protein AKT has also been reported to play important roles in muscle protein homeostasis, function, and metabolism.^21^ Besides increasing protein intake and adequate exercise, a positive effect on muscle health is reported to be exerted by phytochemicals like curcumin or sulforaphane.^22^ Notably, the promoting effect of curcumin on activation of the PI3K/AKT/mTOR pathway has been demonstrated in quadriceps to mitigate exercise fatigue.^23^

Feruloylacetone (FER) is a thermal degradant of curcumin and is present in turmeric-containing dishes after cooking.^24^ Our previous study suggested that supplementation with feruloylacetone (FER) and its demethoxy analog, demethoxyferuloylacetone (DFER), positively impacts high-fat diet (HFD) fed mice by promoting thermogenesis and enhancing the growth of gut microbial short-chain fatty acid (SCFA) producers.^25^ However, the broader health benefits of these curcumin thermal degradants have not been clearly elucidated. Therefore, this study aims to investigate whether FER and DFER might exhibit beneficial or preventive effects on obesity-related glucose intolerance and obesity-associated sarcopenia, as well as to explore the underlying molecular mechanisms.

## 2 Materials and Methods

Sabinsa Corporation (New Jersey, USA) generously supplied feruloylacetone (FER) and demethoxy feruloylacetone (DFER) with a purity exceeding 95%. GLUT2 and GLUT4 were sourced from Proteintech (IL, USA). Antibodies targeting anti-p-Akt, t-Akt, p-IRS-1, t-IRS-1, p-mTOR, t-mTOR, p-PI3K, and t-PI3K, were procured from Cell Signaling Technology (MA, USA), while anti-*p*-Eukaryotic translation initiation factor 4E (eIF4E)-binding protein 1 (4EBP1), t-4EBP1, F-box only protein 32 (FBXO32), p- Ribosomal protein S6 kinase beta-1 (p70S6K), t-p70S6K, and Muscle Ring-Finger Protein-1 (TRIM63) were obtained from ABclonal Biotech Co., Ltd. (China).

### 2.1 In Vivo Study Design

A total of 40 male C57BL/6 mice were procured from the National Laboratory Animal Center in Taipei, Taiwan. The mice were housed in a controlled environment with a temperature maintained at 25 ± 1 °C and a relative humidity of 50%. Ethical guidelines were strictly adhered to in conducting the animal study, and approval was obtained from the Institutional Animal Care and Use Committee (IACUC) of the National Taiwan University (reference number NTU-111-EL-00088). Following a one-week acclimation period, the mice were stratified into four groups, each comprising 8 individuals. A control group (ND) was administered a standard chow diet (Purina 5001, Lab Diet), while the remaining groups were fed a high-fat diet (HFD) comprising 50% of caloric intake from fat. FER and DFER were incorporated into the diet at a concentration of 0.25% (w/w) daily for a period of 16 weeks. Weekly body weight measurements were recorded and, upon completion of the 16 week study period, the mice were euthanized humanely using CO_2_ asphyxiation. Subsequently, their gastrocnemius muscles and organs were weighed, photographed, and preserved at −80 °C for subsequent analysis. The organ index is calculated as the ratio of organ weight to body weight.

A 0.25% (w/w) sample in the diet corresponds to a dosage of 8.75 mg/day for a 30 g mouse (292 mg/kg/day). After conversion, this equates to approximately 1.42 g/day for a 60 kg adult or 146 mg/kg/day for a rat.

### 2.2 Muscle Triglyceride Content

The triglyceride content in gastrocnemius muscles tissue was determined with a triglyceride colorimetric assay kit (10010303, Cayman) following the manufacturer’s protocol. The tissues were weighed, homogenized, and the supernatants were collected for analysis.

### 2.3 Hematoxylin-Eosin (H&E) Staining Procedure

For histopathological analysis, the mice livers and gastrocnemius muscles collected underwent hematoxylin and eosin (H&E) staining to facilitate the visualization of structural details. Initially, harvested tissue samples were fixed in a 10% formalin buffer solution. Following fixation, the tissues were dehydrated, embedded in paraffin, and sectioned into thin slices measuring 3–5 μm in thickness. The tissue sections were deparaffinized using xylene and rehydrated with ethanol/water prior to H&E staining.

### 2.4 Immunohistochemistry (IHC) and Glycogen Staining

IHC staining was carried out using an HRP/DAB IHC Detection Kit (ab236466, Abcam PLC, UK) following the manufacturer’s procedure. GLUT2 and GLUT4 were applied at a dilution of 1:200 and incubated at 4 °C overnight.

Glycogen staining was carried out with a periodic acid Schiff (PAS) stain kit (ab150680, Abcam PLC, UK) according to the manufacturer’s protocol.

### 2.5 Western Blot Procedure

Gastrocnemius muscles tissues were homogenized and subsequently lysed using an ice-cold lysis buffer, followed by incubation on ice for a minimum of 1 h. Posthomogenization, samples underwent centrifugation at 14,000*g* for 1 h at 4 °C. The resulting supernatants were collected and stored at −80 °C until further analysis. Protein lysate concentrations were determined utilizing a Bio-Rad protein assay. For electrophoresis, 25 μg of protein samples were loaded into individual wells and transferred onto PVDF membranes from Merck Millipore Ltd. (Tullagreen, County Cork, Ireland). Following transfer, membranes underwent a blocking procedure (with blocking agent containing 20 mM Tris-base, 137 mM NaCl, 1% BSA (w/v), 1% Tween 20 and 0.1% sodium azide), followed by overnight incubation with primary antibodies. To ensure optimal antibody binding and removal of unbound antibodies, membranes were subjected to multiple washes with a solution containing 0.2% phosphate-buffered saline Tween 20 (TPBS), both before and after the application of secondary antibodies. Protein band visualization was achieved through chemiluminescence (ECL, Merck Millipore Ltd.), and densitometry analysis of the bands was conducted using ImageJ imaging software. GAPDH served as an internal control for Western blotting.

### 2.6 Fasting Glucose, Oral Glucose Tolerance Test (OGTT), and HOMA-IR

Fasting blood glucose testing was conducted on the mice at week 15 of the study. Following a 10 h fast, blood samples were obtained via tail nick to determine glucose levels. Oral glucose tolerance testing (OGTT) was performed similarly to fasting blood glucose testing, with the additional step of administering extra glucose at a dosage of 2 g/kg of body weight through oral administration after an overnight fast. Glycemia testing was conducted before the administration of glucose at the zero-time point, followed by measurements at 30, 60, 90, 120, and 180 min after glucose administration.

Fasting insulin was determined using an Insulin ELISA kit (10-11136-01, Mercodia, Sweden) following the manufacturer’s procedure. HOMA-IR was calculated using the following equation.
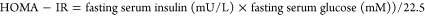


### 2.7 Gut Microbiota Analysis

Colonic feces were collected and stored at −80 °C. Fecal microbial DNA extraction and purification were performed using the innuPREP Stool DNA Isolation Kit. Primers with a 5′ buffer sequence (GCATC) and a 5′ phosphate modification were designed for genomic DNA amplification. HiFi reads with a predicted accuracy (Phred scale) of 30 were generated using the PacBio Sequel IIe instrument in circular consensus sequence (CCS) mode. For statistical analysis, the significance of all species among groups at various taxonomic levels was assessed using differential abundance analysis with a zero-inflated Gaussian (ZIG) log-normal model, implemented in the “fitFeatureModel” function of the Bioconductor metagenomeSeq package. Additionally, Welch’s *t* test was performed using the stat package in R. Statistically significant biomarkers were identified using LEfSe analysis. LEfSe employs an algorithm that performs the nonparametric Kruskal–Wallis test and Wilcoxon rank-sum test to identify bacterial taxa with significant differences in relative abundance between control and experimental groups. LEfSe applies linear discriminant analysis (LDA) to the significantly different bacterial taxa to assess the effect size of each differentially abundant taxon. In this study, taxa with an LDA score (log10) > 3 were considered significant.

### 2.8 In Vitro Study

The C2C12 murine myoblast cell line (ATCC) was used in the in vitro study. The cells were cultured in DMEM supplemented with 10% heat-inactivated FBS (Gibco). Initially, the cells were seeded at a density of 1 × 10^4^ cells per well and incubated for 12 h. The medium was then replaced with DMEM supplemented with 5% horse serum to induce differentiation. The medium was changed every 2 days, and photographs were taken to observe the status of the cells during differentiation. Differentiation was completed on day 8, and the images of the differentiated C2C12 myotubes are shown in Figure S1. Dexamethasone (DEX, 100 μM) was used as the inducer to mimic muscle atrophy, while TNF-α (100 ng/mL) or LPS (100 ng/mL) were used to induce insulin resistance.

#### 2.8.1 Glucose Uptake Analysis

The capability of glucose uptake was assessed using 2-NBDG (N13195, Invitrogen), and flow cytometry (CytoFLEX, Beckman Coulter) was employed for the analysis. Differentiated myotubes were cultured in DMEM supplemented with 1% FBS and glucose (1 g/L), and treated with inducers and samples for 24 h. The cells were then washed with PBS and incubated in Krebs-Ringer Bicarbonate Buffer with insulin (100 nM) for 30–40 min. Following this, 2-NBDG was added, and the cells were allowed to uptake 2-NBDG for 1 h. After 1 h, the cells were trypsinized, centrifuged, and washed once with PBS. Following a second centrifugation, the cells were resuspended in PBS, and the cellular 2-NBDG uptake was determined using flow cytometry.

#### 2.8.2 Western Blot for In Vitro Study

For Western blot analysis, differentiated myotubes were cultured in DMEM supplemented with 1% FBS and glucose (1 g/L), and treated with inducers and samples for 24 h. Following treatment, the medium was replaced with DMEM supplemented with 10% heat-inactivated FBS, and insulin and PI3K inhibitor LY294002 (80 μM) were added. The cells were collected after 6 h for protein analysis.

### 2.9 Statistical Analysis

Results are presented as mean ± standard error (S.E.). Significant differences between groups were determined using One-way ANOVA (ANalysis Of VAriance) with posthoc Tukey HSD (Honestly Significant Difference) Test. Significant differences between groups were determined using the Student’s *t* test. In this study, a *p*-value of <0.05 was considered statistically significant.

## 3 Results

### 3.1 Both FER and DFER Significantly Reverse the Reduction in Gastrocnemius Weight Induced by HFD Feeding

**Figure 1:**
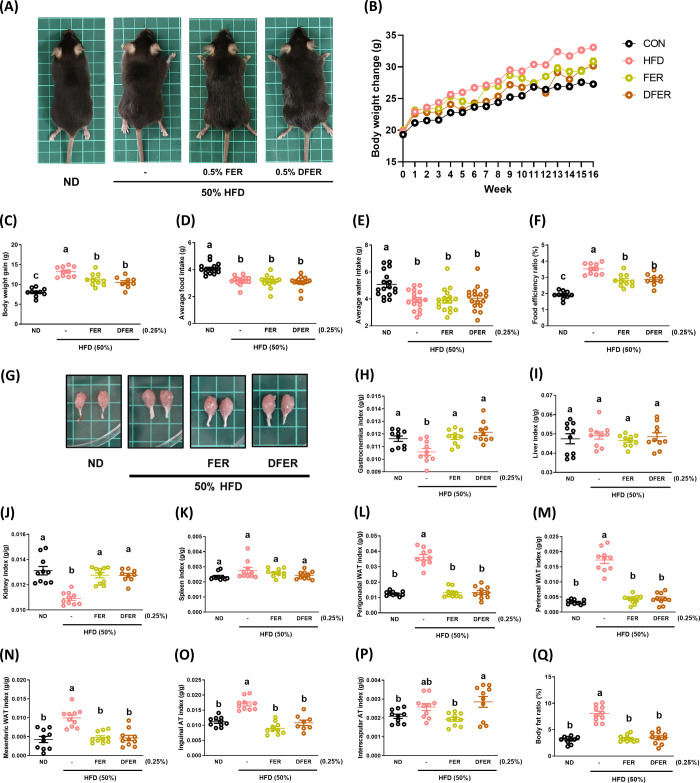
Both FER and DFER significantly reverse the reduction in gastrocnemius weight induced by HFD feeding. (A) Representative appearance of the mice from each group, (B) body weight change over 16 weeks, (C) body weight gain after 16 weeks, (D) average food intake, (E) average water intake, (F) food efficiency ratio (%), (G) representative appearance of gastrocnemius, (H) gastrocnemius index, (I) liver index, (J) kidney index, (K) spleen index, (L) perigonadal WAT index, (M) perirenal WAT index, (N) mesenteric WAT index, (O) inguinal AT index, (P) interscapular AT index, and (Q) body fat ratio (%). All data are presented as mean ± S.E., *n* = 10. Different lowercase letters indicate significant differences among groups, as determined by ANOVA followed by Tukey’s post hoc test.

After being fed a 50% high-fat diet (HFD), the HFD group exhibited a significant increase in body weight. However, supplementation with 0.25% FER or DFER in the diet effectively mitigated this increase in body weight (Figure 1A–C). The average food intake analysis indicated that the weight changes in the supplemented group were not due to changes in appetite (Figure 1D), and the food efficiency ratio (Figure 1F) further supported the positive impact of these curcuminoid degradants on diet-induced obesity. The effects of FER and DFER supplementation on various organs are depicted in Figure 1G–P. The results reveal that HFD feeding significantly reduced the weights of the gastrocnemius muscle and kidney, while markedly increasing the weights of adipose tissues. Surprisingly, both FER and DFER supplementation significantly prevented the loss of weight of the gastrocnemius and kidney, and reduced the weight gains in adipose tissues.

### 3.2 Supplementation with FER and DFER Improves Hyperglycemia and Insulin Resistance in HFD Mice

**Figure 2:**
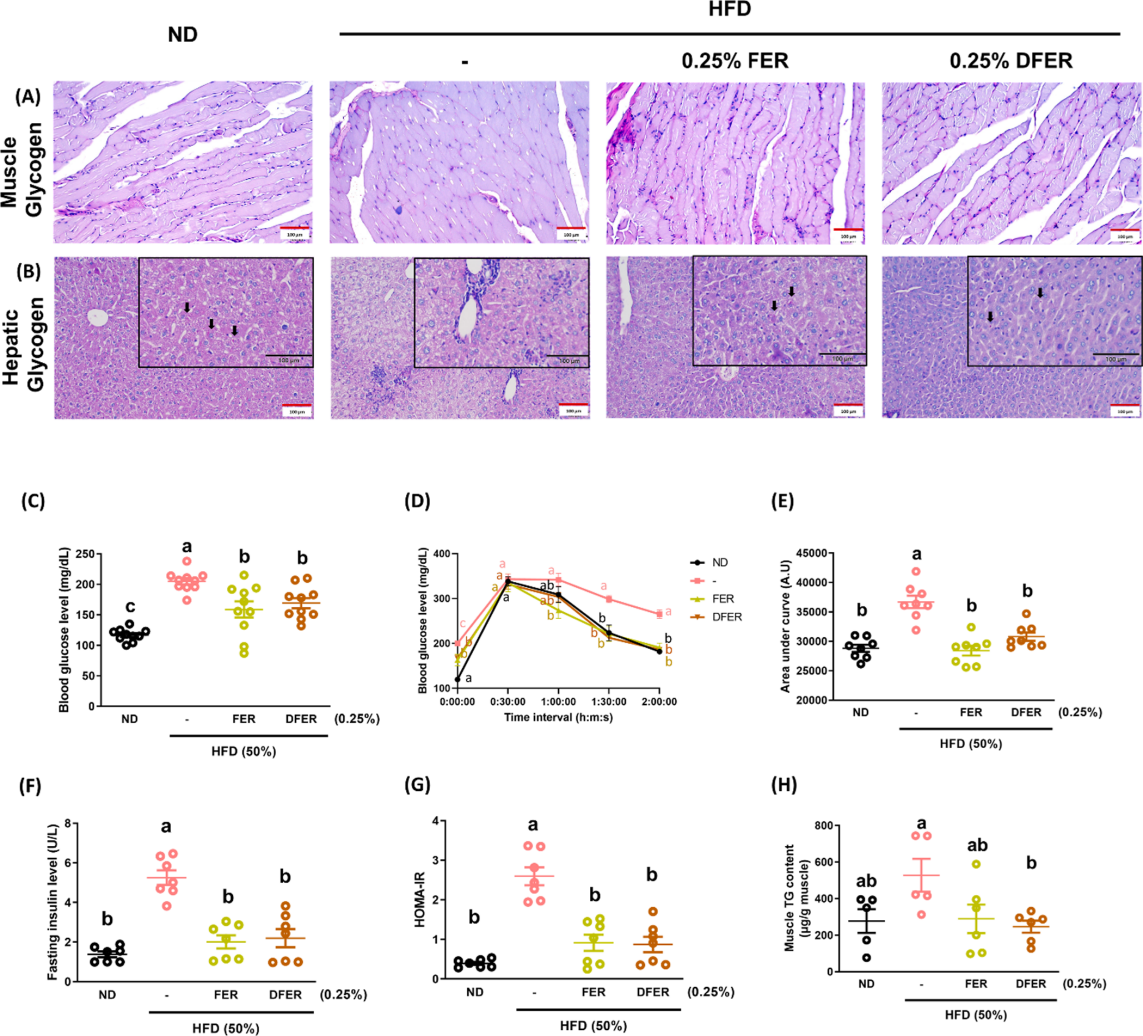
Both FER and DFER significantly ameliorate glucose homeostasis in HFD-fed mice. PAS staining of glycogen in (A) gastrocnemius tissue and (B) liver tissue. Black arrows indicate stained glycogens with pinkish staining. All images were viewed at 200× magnification (scale bar = 100 μm). (C) Fasting blood glucose level (*n* = 10), (D) oral glucose tolerance test results (*n* = 8), (E) area under the curve of D (*n* = 8), (F) fasting insulin level (*n* = 7), (G) HOMA-IR (*n* = 7), and (H) muscle triglyceride (TG) content (*n* = 5–6). All data are presented as mean ± SE. Different lowercase letters indicate significant differences among groups, as determined by ANOVA followed by Tukey’s post hoc test.

Muscle and hepatic glycogen were visualized using PAS staining. The area with purple staining (indicating stained glycogen) was lower in the HFD group across all samples (Figure 2A,B). A significant increase in fasting blood glucose was also observed in the HFD-fed group, which was prevented by supplementation (Figure 2C). The results of the OGTT showed a more gradual decrease in the curve in the HFD group, while both supplemented groups were able to recover blood glucose levels similar to the control group after 90 min (Figure 2D). This result was further supported by the area under the curve (Figure 2E). The fasting insulin level exhibited a pattern similar to that of fasting glucose (Figure 2F), and the HOMA-IR was calculated to evaluate insulin resistance (Figure 2G). Muscle triglyceride (TG) content was determined using an ELISA kit, and the results show that DFER effectively reduced the accumulation of TG in muscle (Figure 2H).

**Figure 3:**
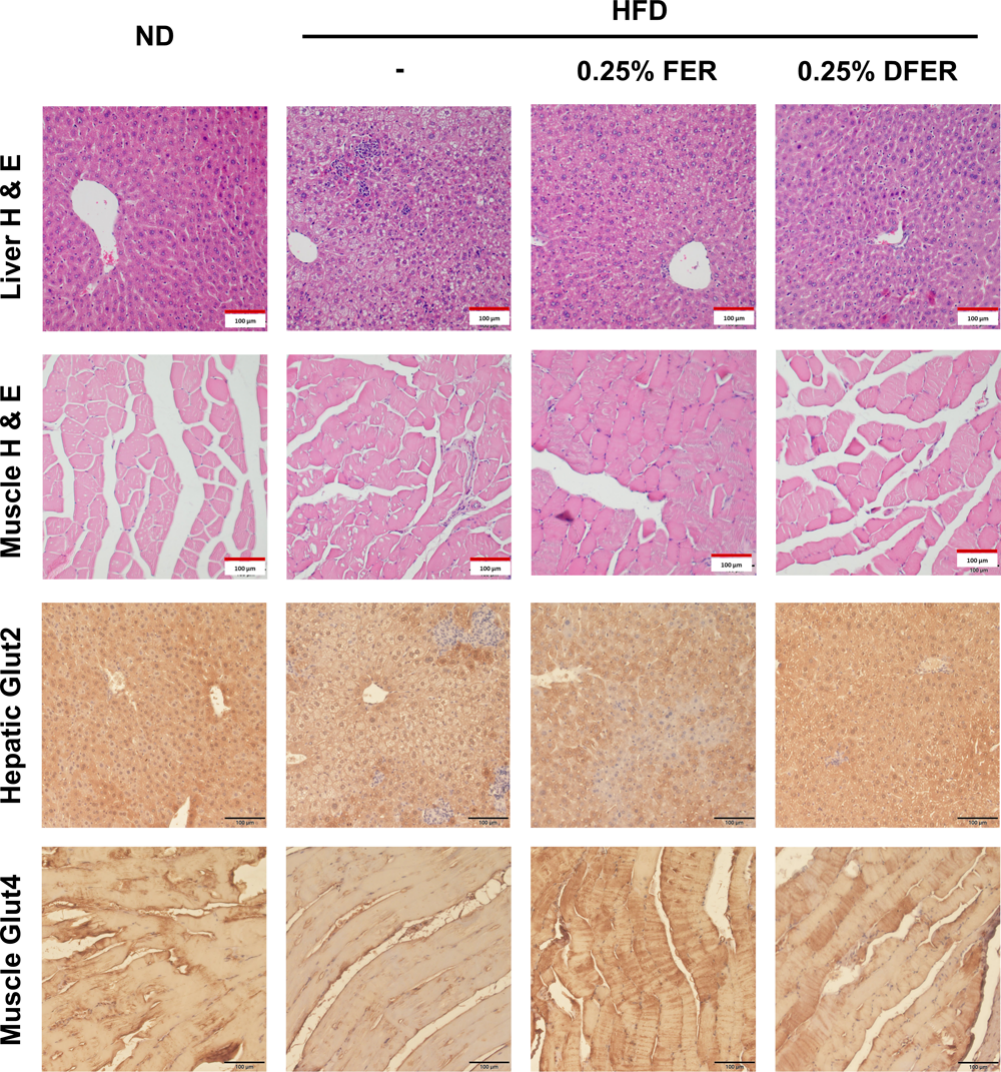
Effect of FER and DFER on the liver and gastrocnemius tissues of HFD-fed mice. H&E staining of liver and gastrocnemius tissue sections, and IHC staining of Glut2 in liver and Glut4 in gastrocnemius tissue sections. All images were viewed at 200× magnification (scale bar = 100 μm).

The effects of HFD feeding on hepatic and muscle histology are presented in Figure 3. In the HFD group, vesicles caused by lipid accumulation were observed and these were reduced by FER and DFER supplementation. Additionally, infiltration of immune cells was found in both liver and muscle tissues in the HFD group. Muscle cells were irregularly arranged, and the muscle fiber area was comparatively inconsistent. Supporting the fasting blood glucose and HOMA-IR results, the expressions of glucose transporter proteins GLUT2 and GLUT4 in different groups exhibited a noticeable reduction (indicated by brownish stained spots) in the liver and muscle sections of the HFD group, and this reduction was reversed by supplementation.

### 3.3 Amelioration of Hyperglycemia in the Supplemented Groups Is Partially Attributed to the PI3K/Akt Pathway

**Figure 4:**
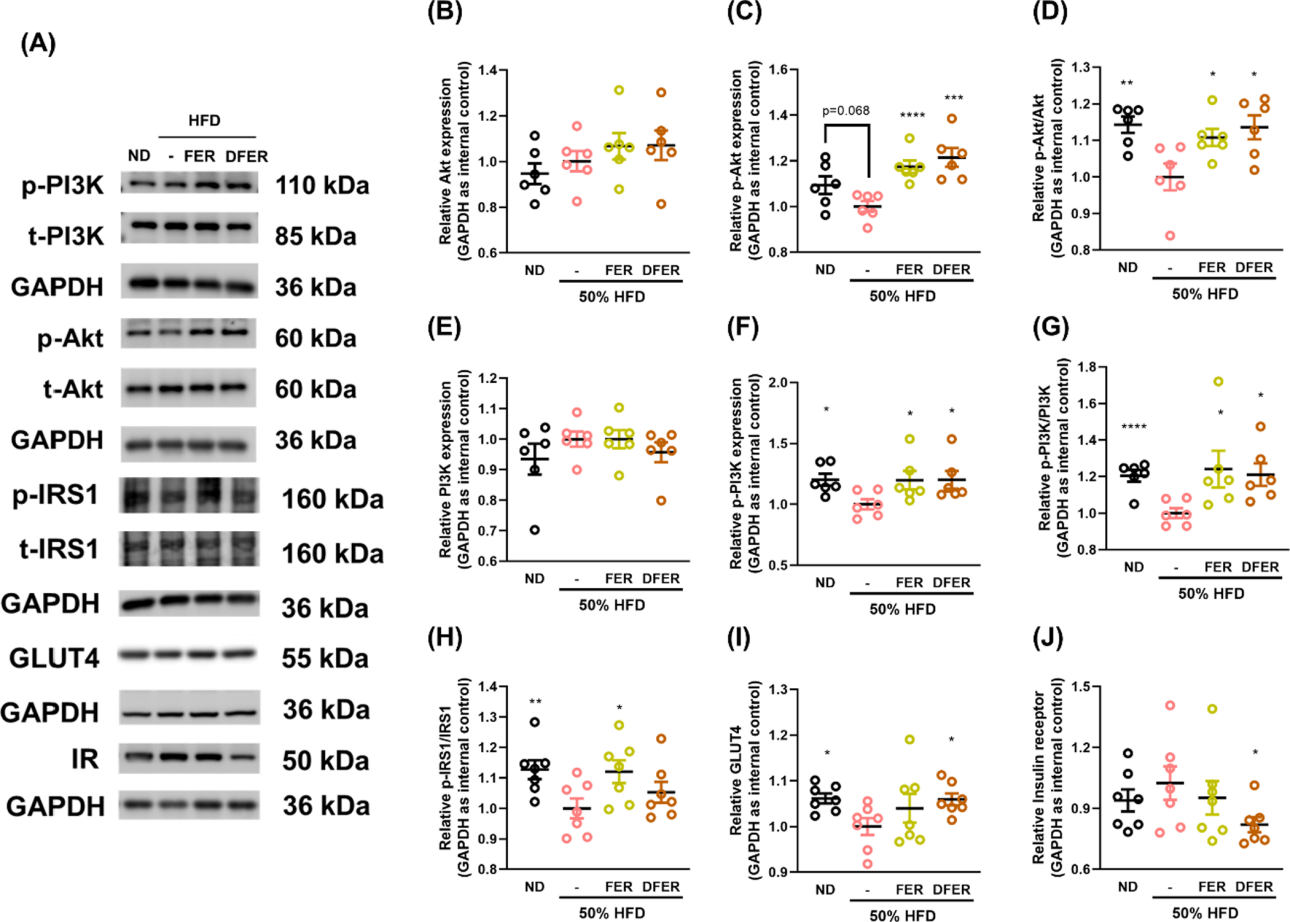
Both FER and DFER ameliorate glucose homeostasis in HFD-fed mice possibly via PI3K/Akt signaling pathway. (A) Representative images of Western blots. Relative expression of (B) Akt, (C) p-Akt, (D) p-Akt/Akt, (E) PI3K, (F) p-PI3K, (G) p-PI3K/PI3K, (H) p-IRS/IRS, (I) GLUT4, and (J) insulin receptor (IR) in gastrocnemius tissue. GAPDH was used as the internal control. All data are presented as mean ± SE (*n* = 6–8). Symbols (*), (**), (***), and (****) indicate significant differences compared to the HFD group, with *p*-values less than 0.05, 0.01, 0.005, and 0.001, respectively, determined by Student’s *t* test.

To confirm the underlying mechanism contributing to the amelioration of insulin resistance and hyperglycemia, Western blot analysis was conducted. Significant reductions in the ratios of p-Akt/Akt (Figure 4A–D) and p-PI3K/PI3K (Figure 4A,F–H) were observed in the HFD group compared to the control group. However, FER and DFER supplementation increased the phosphorylation levels of these proteins. Additionally, the phosphorylation level of their upstream protein IRS was significantly reduced in the HFD group but elevated in the FER group. The level of GLUT4 was also significantly reduced in the HFD group, and this reduction was reversed by DFER supplementation. Furthermore, the insulin receptor (IR) was significantly reduced in the DFER group compared to the HFD group. Our results suggest that the ameliorative effects of FER and DFER on hyperglycemia and insulin resistance might not be completely dependent on the activation of the Akt/PI3K pathway. Moreover, it was suspected that weight loss of the gastrocnemius tissue in the HFD group might contribute to the homeostasis of blood glucose. Therefore, the mechanism of protein synthesis and degradation was further investigated.

### 3.4 FER and DFER Supplementation Promote Protein Synthesis and Inhibit Degradation in Muscle Tissue

**Figure 5:**
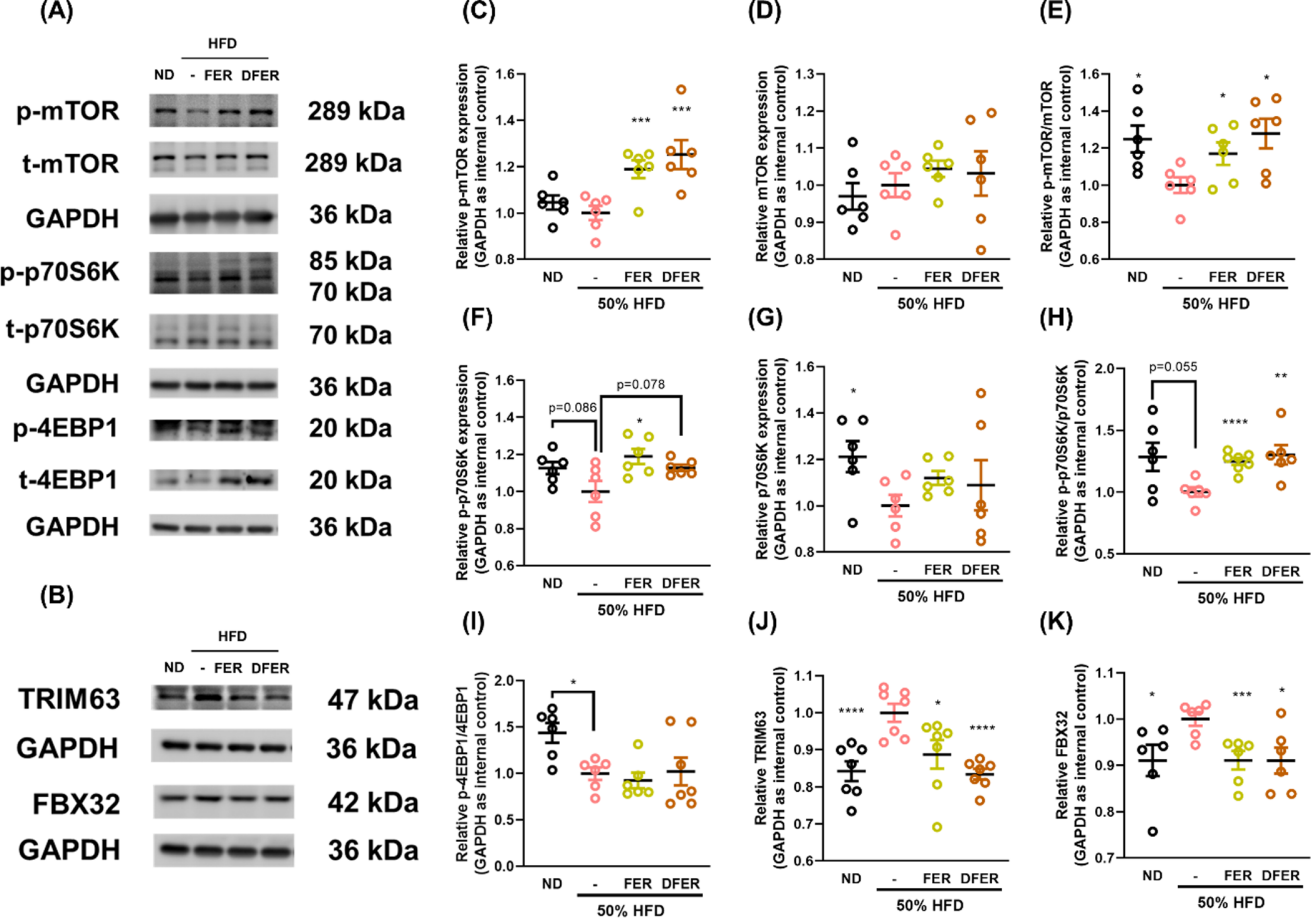
Both FER and DFER alleviate muscle atrophy in HFD-fed mice possibly by enhancing protein synthesis and suppressing protein degradation. (A, B) Representative images of Western blots. Relative expression of (C) p-mTOR, (D) mTOR, (E) p-mTOR/mTOR, (F) p-p70S6K, (G) p70S6K, (H) p-p70S6K/p70S6K, (I) p-4EBP1/4EBP1, (J) TRIM63, and (K) FBX32 in gastrocnemius tissue. GAPDH was used as the internal control. All data are presented as mean ± SE (*n* = 6–8). The symbols (*), (**), (***), and (****) indicate significant differences compared to the HFD group, with *p*-values less than 0.05, 0.01, 0.005, and 0.001, respectively, determined by Student’s *t* test.

To confirm that FER and DFER supplementation contributes to protein synthesis in muscle tissue, the phosphorylation of mTOR, p70S6K, and 4E-BP1 was determined. Our results show that both supplementations effectively enhanced the phosphorylation of the mTOR/p70S6K pathway (Figure 5A,C–H) but had no effect on 4E-BP1 phosphorylation (Figure 5A,I). Additionally, significant reductions in TRIM63 and FBX32 levels suggested protein degradation in the muscle tissues of HFD mice. Both FER and DFER significantly suppressed TRIM63 and FBX32, indicating an alleviation of protein degradation in muscle tissue (Figure 5B,J,K).

To confirm our findings, an in vitro study was designed using the C2C12 myoblast cell line. C2C12 cells were treated with 5% horse serum for 8 days to induce differentiation into myotubes. The medium was changed every 2 days, and images documenting the differentiation process are presented in Figure S1A. At the start, cells were seeded at two different densities; however, no observable differences in the differentiation rate were noted between the densities. Therefore, 1 × 10^4^ cells/well was selected for subsequent analyses. TNF-α and dexamethasone (DEX) were used as inducers in this study, but no noticeable effects on the appearance of the cells were observed.

In Figure S2A–C, differentiated myotubes were treated with DEX for 24 h to induce protein degradation, followed by pretreatment with insulin and subsequent exposure to 2NBDG to assess glucose uptake capability. Figure S2A shows that DEX significantly reduced 2NBDG uptake in insulin-pretreated myotubes, confirming the successful establishment of the insulin resistance model. Figure S2B,C demonstrate that treatment with FER and DFER at a concentration of 40 μM significantly reversed insulin resistance. To determine whether the enhanced glucose uptake capability was partially attributed to the suppressive effects of FER and DFER on protein degradation, Western blot analysis was performed. As shown in Figure S2D–F,G–I, both FER and DFER significantly reduced markers of muscle atrophy, with DFER exhibiting a more pronounced effect.

Additionally, the ability of FER and DFER to promote glucose uptake in uninduced myotubes was evaluated. As shown in Figures S2E,F and S3A,B, both FER and DFER enhanced 2NBDG uptake, regardless of insulin presence. Western blot results further revealed that FER and DFER treatment for 6 h could induce the phosphorylation of PI3K (Figure S3C,D). To further confirm the role of FER and DFER to improve DEX-treated myotubes, PI3K inhibitor LY294002 was employed. However, there was no effect on glucose uptake capability when LY294002 was treated at the same time with FER and DFER for 24 h, with or without insulin treatment (Figure S3G–J). The result indicated that PI3K activation was not the major pathway for FER and DFER to alleviate DEX-induced protein degradation.

**Figure 6:**
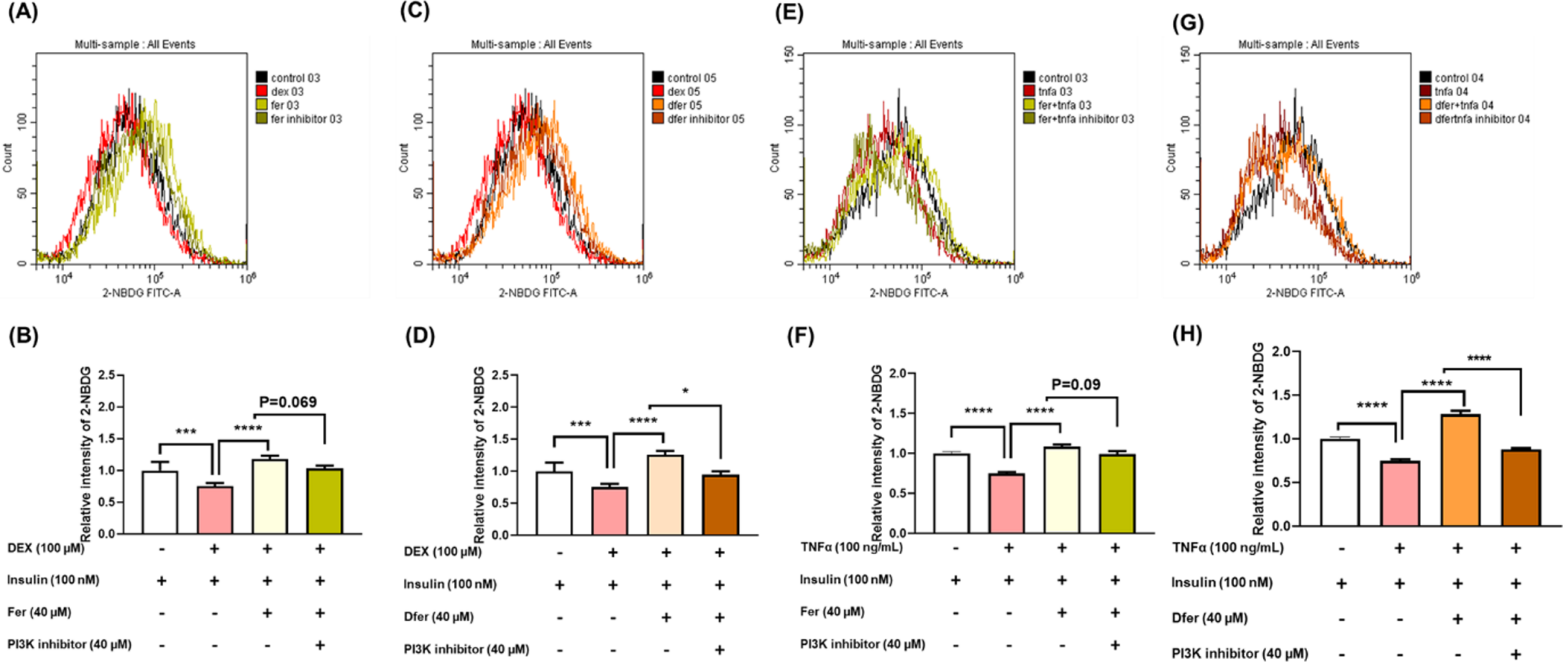
FER and DFER promote insulin sensitivity in DEX- or TNF-α-induced myotubes via PI3K-mediated pathway. (A) Representative flow cytometry image showing 2NBDG uptake after 24 h treatment with DEX, followed by treatment with PI3K inhibitor for 1 h, and intervention with insulin and FER for 40 min. (B) Quantification of 2NBDG uptake in DEX-induced myotubes after treatment with PI3K inhibitor and intervention with insulin and FER. (C) Representative flow cytometry image showing 2NBDG uptake after 24 h treatment with DEX, followed by treatment with PI3K inhibitor for 1 h, and intervention with insulin and DFER for 40 min. (D) Quantification of 2NBDG uptake in DEX-induced myotubes after treatment with PI3K inhibitor and intervention with insulin and DFER. (E) Representative flow cytometry image showing 2NBDG uptake after 24 h treatment with TNF-α, followed by treatment with PI3K inhibitor for 1 h, and intervention with insulin and FER for 40 min. (F) Quantification of 2NBDG uptake in TNF-α-induced myotubes after treatment with PI3K inhibitor and intervention with insulin and FER. (G) Representative flow cytometry image showing 2NBDG uptake after 24 h treatment with TNF-α, followed by treatment with PI3K inhibitor for 1 h, and intervention with insulin and DFER for 40 min. (H) Quantification of 2NBDG uptake in TNF-α-induced myotubes after treatment with PI3K inhibitor and intervention with insulin and DFER.

To further confirm the effects of FER and DFER on insulin sensitivity and glucose uptake, myotubes were treated with either DEX or TNF-α for 24 h. The cells were then pretreated with insulin and PI3K inhibitors, followed by intervention with FER and DFER. Interestingly, both FER and DFER enhanced the insulin sensitivity of DEX- or TNF-α-induced cells, but this improvement was diminished upon the addition of the PI3K inhibitor. These findings suggest that FER and DFER enhance insulin sensitivity and glucose uptake in insulin-resistant (Figure 6A,B) and atrophic cells (Figure 6C,D) through a PI3K-mediated pathway.

Lastly, the effects of FER and DFER on the activation of the PI3K/Akt/mTOR pathway were evaluated through Western blot analysis (Figure S4). The results demonstrated that both FER and DFER effectively reversed the TNF-α-induced deactivation of the PI3K/Akt/mTOR pathway. However, this effect was significantly diminished upon the addition of PI3K inhibitors. These findings indicate that FER and DFER promote the activation of the PI3K/Akt/mTOR pathway and prevent protein degradation.

### 3.5 Recomposition of Gut Microbiota by FER and DFER May Contribute to Hyperglycemia and Insulin Resistance Improvement

Chronic inflammatory responses accompanied by the infiltration of immune cells in various tissues have been associated with the incidence of insulin resistance and diabetes.^26^ Our results show that proinflammatory cytokines and chemokines, such as Interleukin-6 (IL-6), Tumor Necrosis Factor Alpha (TNF-α), Interferon gamma (IFN-γ), and Monocyte chemoattractant protein-1 (MCP-1), were elevated in the muscle tissues of HFD-fed mice. However, these elevations were significantly suppressed in both sample groups (Figure 6A–D). It has been suggested that inflammatory responses are associated with the presence of certain bacteria.^27^ Therefore, the gut microbial composition was analyzed.

**Figure 7:**
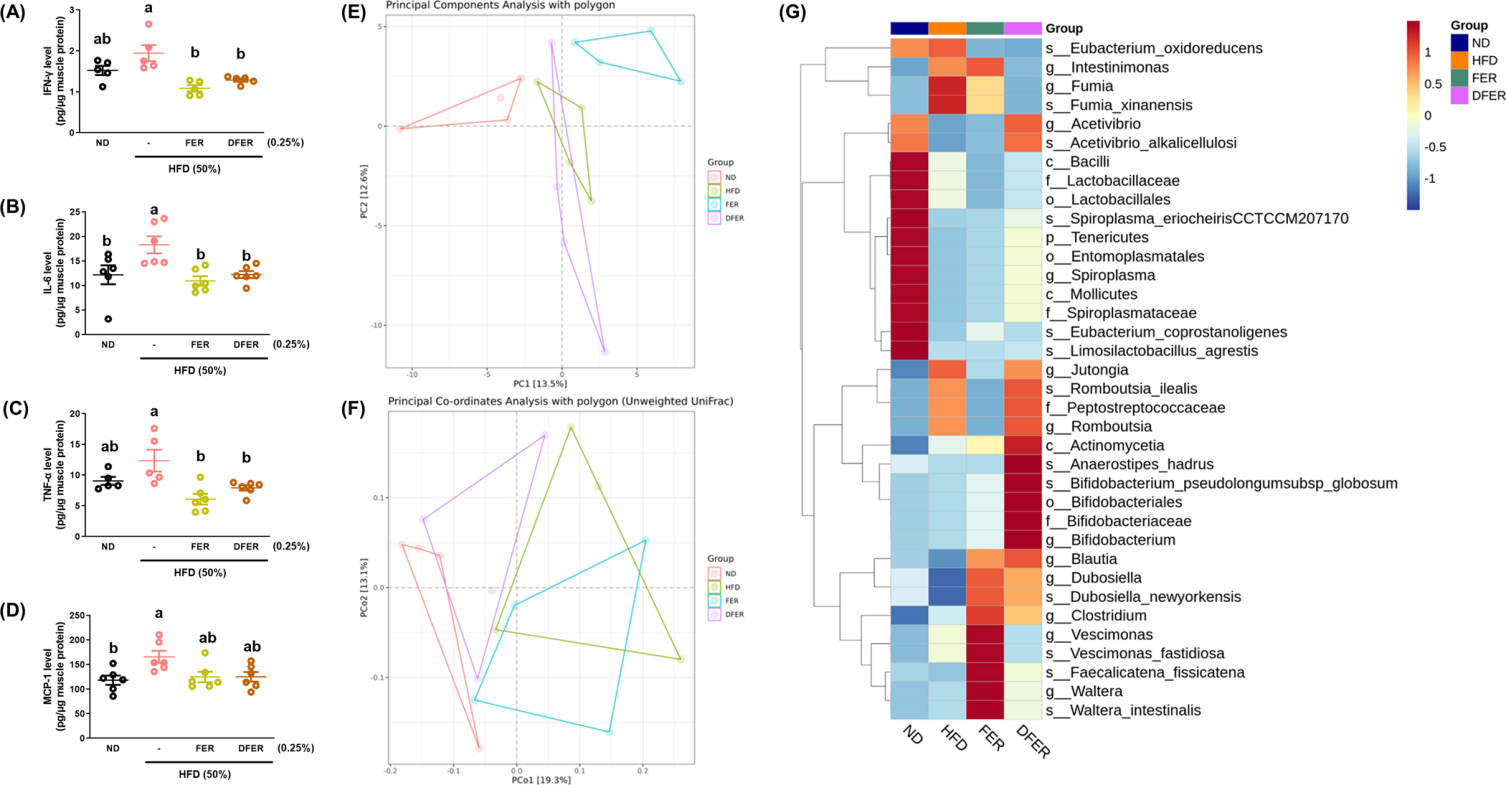
Both FER and DFER reduce pro-inflammatory responses possibly by improving gut microbial dysbiosis induced by HFD-feeding. (A) IFN-γ, (B) IL-6, (C) TNF-α, and (D) MCP-1 levels in gastrocnemius tissue. (E) PCA plots and (F) PCoA plots of gut microbiota. (G) Heat map of the linear discriminant analysis (LDA) effect size (LEfSe).

The result of the principal components analysis (PCA) indicates that while some of the most abundant species were similar between the HFD and DFER groups, the FER group had a distinct composition (Figure 7E). However, by employing principal coordinates analysis (PCoA), the FER group showed stronger similarity to the HFD group than to the DFER and ND groups (Figure 7F). The result of β-diversity was supported by the plots of constrained analysis of principal coordinates and PLS-DA, showing distinct gut microbial composition among the experimental groups (Figure S5A,B).

The result of LefSe analysis shows that HFD feeding led to a decreased abundance of *Acetivibrio alkalicellulosi*, *Lactobacillaceae*, *Spiroplasma eriocheiris* (CCTCC M 207170), *Eubacterium coprostanoligenes*, and *Limosilactobacillus agrestis*. Among these, the abundance of *A. alkalicellulosi* and *S. eriocheiris* could be preserved by DFER, while FER could only preserve the abundance of *E. coprostanoligenes*. In contrast, HFD feeding led to an elevation in the abundances of *Jutongia* and *Romboutsia ilealis*, which were suppressed by FER but not DFER. Additionally, a significant elevation in the abundances of *Intestinimonas* and *Fumia xinanensis* was found in the HFD group, which was suppressed by supplementation with FER. Comparatively, supplementation with both FER and DFER facilitated the growth of Blautia, *Dubosiella newyorkensis*, *Clostridium viride*, *Faecalicatena fissicatena*, and *Waltera intestinalis*. Furthermore, there were some species whose abundance was found exclusively in either the FER or DFER groups. For example, *Anaerostipes hadrus* and *Bifidobacterium pseudolongum* subsp. *globosum* were found in the DFER group, while *Vescimonas fastidiosa* was found in the FER group (Figure 7G). The correlation network of genus and species is presented in Figure S5C,D, showing the correlation between gut microbes. For instance, *B. intestinalis*, *D. newyorkensis*, *F. fissicatena*, and *C. viride*, whose abundances are found to be elevated in the FER and DFER groups, are positively correlated with each other (Figure S5D).

The purpose of LEfSe is to identify biomarkers that are differentially abundant between compared groups. By employing Statistical Analysis of Metagenomic Profiles (STAMP), differences in taxonomic profiles between sample groups can be observed, with *q*-values used to reduce false positives. The biomarkers found in LEfSe were also identified in STAMP, but more species were identified (Table 1). These results provide more information about the gut microbial composition modulated by supplementation of FER and DFER after HFD feeding.

**Table 1:** Comparison of Gut Microbial Abundance between Experimental Groups Using STAMP

taxonomy (species)	logFC	SE	*q-*values
ND vs HFD			
Bacteroides_acidifaciens	–4.59732	0.951579	7.03 × 10^–05^
Christensenella_hongkongensis	–1.70147	0.544325	0.026595
Clostridium_polynesiense	–3.22774	0.674475	7.03 × 10^–05^
Eubacterium_coprostanoligenes	–4.51256	0.613724	1.6 × 10^–11^
Fumia_xinanensis	2.328845	0.538261	0.0005
Jutongia_hominis	3.135731	0.862887	0.005116
Limosilactobacillus_agrestis	–3.51992	0.471693	1.4 × 10^–11^
Massiliimalia_timonensis	1.671835	0.434176	0.002777
Oscillibacter_valericigenes	1.665965	0.649961	0.015846
Provencibacterium_massiliense	1.221168	0.507429	0.003336
Romboutsia_ilealis	3.096486	0.937795	0.002443
Solibaculum_mannosilyticum	3.168204	0.839858	7.03 × 10^–05^
Spiroplasma_eriocheiris CCTCC M 207170	–3.51025	0.895656	0.026595

taxonomy (species)	logFC	SE	*q*-values
HFD vs FER			
Faecalicatena_fissicatena	2.990115	0.610487	9.69 × 10^–07^
Guopingia_tenuis	3.201364	0.713649	7.26 × 10^–06^
Luxibacter_massiliensis	–3.01729	0.741119	4.68 × 10^–05^

taxonomy (species)	logFC	SE	*q*-values
HED vs DFER			
Anaerocolumna_cellulosilytica	2.326959	0.723133	0.001291
Anaerostipes_hadrus	4.148499	0.795212	1.82 × 10^–07^
Blautia_intestinalis	1.957538	0.670539	0.003508
Blautia_producta ATCC 27340 = DSM 2950	2.168922	0.740468	0.003399
Dubosiella_newyorkensis	2.374505	0.772768	0.002121
Faecalicatena_fissicatena	1.754515	0.591624	0.003021
Fumia_xinanensis	–2.17557	0.569038	0.000132
Lacrimispora_indolis	–1.89285	0.644611	0.00332
Roseburia_hominis A2–183	2.093277	0.612236	0.000628
Roseburia_inulinivorans DSM 16841	1.990279	0.590473	0.00075
Roseburia_porci	2.162057	0.618537	7.99 × 10^–06^
Spiroplasma_eriocheiris CCTCC M 207170	2.151877	0.698372	0.002061

Our previous study found that FER and DFER facilitated the growth of gut microbial SCFA producers and significantly increased fecal SCFA levels.^25^ To confirm the benefits of these SCFAs, an in vitro study was designed (Figure S6). After induction with lipopolysaccharides (LPS, 100 ng/mL) for 24 h, cellular 2NBDG uptake levels significantly increased, which might be attributed to the induction of GLUT1 by LPS.^28^ Our results showed that acetic acid, propionic acid, and valeric acid effectively reversed the abnormal glucose uptake induced by LPS. To validate these findings, we used TNF-α to induce glucose uptake.^29^ The results demonstrated that all the SCFAs significantly inhibited the glucose uptake induced by TNF-α (Figure S6E,F). In summary, FER and DFER could promote the production of gut microbial SCFAs, which effectively reverse abnormal glucose uptake induced by LPS and TNF-α.

To confirm the role of SCFAs, a correlation analysis was performed between fecal short-chain fatty acids (SCFAs) and key physiological parameters, including muscle weight, fasting blood glucose, fasting insulin, HOMA-IR, and inflammatory markers. The analysis revealed significant positive correlations (*p* < 0.05) between fecal propionate, butyrate, valerate, total SCFAs, and the muscle index, suggesting that SCFAs may have a protective role against muscle atrophy (Figure S7). Furthermore, acetate and valerate demonstrated significant negative correlations with fasting blood glucose levels (*p* < 0.05). However, no significant correlations were observed between SCFAs and fasting insulin levels. For HOMA-IR, acetate and valerate displayed a negative correlation trend, but the differences did not reach statistical significance (Figure S8). Lastly, the relationship between SCFAs and inflammatory markers in muscle tissue was examined. The results showed that fecal acetate, propionate, and butyrate were negatively correlated with several pro-inflammatory markers in muscle tissue, including TNF-α, IL-6, and MCP-1, suggesting that these SCFAs may help mitigate inflammation and improve insulin sensitivity. In contrast, valerate did not exhibit a clear correlation with these inflammatory markers (Figure S9).

## 4 Discussion

Our previous study demonstrated that curcuminoid degradants FER and DFER exhibit beneficial effects on obesity by regulating lipid metabolism, promoting thermogenesis, and modulating gut microbiota. Therefore, this study aimed to investigate the broader health benefits of FER and DFER, including their effects on obesity-related sarcopenia and insulin resistance in muscle tissue. Our results show that a high-fat diet (HFD) led to a significant reduction in the gastrocnemius index, which could be mitigated by supplementation with FER and DFER at 0.25% w/w in the daily diet. Additionally, reductions in glycogen content and increases in fasting blood glucose, fasting insulin level, HOMA-IR, and muscle triglyceride levels caused by obesity were ameliorated by the supplementation. Hyperglycemia and muscle loss were alleviated by FER and DFER supplementation, possibly via activation of the IRS/PI3K/Akt pathway, enhancing protein synthesis, and inhibiting protein degradation.

Chronic inflammation associated with obesity is suggested to be a major factor contributing to insulin resistance. Increasing evidence shows that proinflammatory responses in intermyocellular and perimuscular adipose tissue adversely regulate myocyte metabolism.^30^ In our study, increased levels of IL-6, TNF-α, MCP-1, and IFN-γ were observed in muscle tissues (Figure 6A–D) after 12 weeks of HFD feeding. These increases are associated with adverse responses in glucose homeostasis (Figure 2C–G). Additionally, previous studies suggest that fat accumulation in muscle can impair energy metabolism, including glucose homeostasis, leading to catabolism and atrophy in skeletal muscle tissue.^31^ In our study, the reduction in muscle mass index (Figure 1G–H) and increased TG content in muscle tissue (Figure 2H) in obese mice were consistent with previous findings. Dietary supplementation with the curcuminoid degradants FER and DFER at 0.25% significantly prevented muscle mass loss, possibly by ameliorating obesity and reducing pro-inflammatory responses. In a previous study, supplementation with curcumin (0.4%) effectively reduced lipopolysaccharide (LPS)-induced IL-6 secretion, and it was speculated that the upregulation of mTOR by curcumin might contribute to this effect.^32^ In our study, both FER and DFER significantly increased the phosphorylation of mTOR that was reduced in the HFD group (Figure 5A–E). This increase in mTOR phosphorylation could be associated with the decreased pro-inflammatory response.

The PI3K/Akt pathway is one of the most important anabolic signaling pathways, stimulating mTOR and resulting in muscle protein synthesis. The activation of this pathway is regulated by insulin and insulin-like growth factor 1 (IGF-1).^22^ It has been suggested that reduced skeletal muscle mass is associated with insulin resistance in adults. Therefore, promoting protein synthesis in skeletal tissue could be a potential strategy to address insulin resistance. Improving insulin resistance could, to some extent, prevent atrophy in skeletal muscle tissue.

A recent review indicates that curcumin supplementation can effectively prevent muscle degeneration, protect mitochondrial function, and reduce oxidative stress and inflammation, thereby preventing the pathogenesis of sarcopenia.^33^ It was demonstrated by Sani et al. that, among various tested curcuminoids, curcumin could significantly inhibit the specific markers of muscle atrophy, Atrogin-1, and MuRF1, possibly by enhancing phosphorylation of Akt in the mTOR signaling pathway in a dexamethasone-induced atrophy differentiation in a C2C12 cell line.^34^ Moreover, ferulic acid, a degradant of curcumin, has been shown to positively affect muscle fiber type formation in C2C12 cells^35^ and promote the growth of fast skeletal muscle in zebrafish.^36^ In our study, supplementation with FER and DFER activated the PI3K/Akt/mTOR pathway leading to phosphorylation of p70S6K, which suggests enhanced muscle protein synthesis (Figure 5F–H). Additionally, muscle atrophy markers Fbx32 and TRIM63 were increased in the HFD groups but were prevented by the supplementations, indicating that these compounds could also inhibit muscle protein degradation. A previous study suggested that pro-inflammatory cytokines like IL-1β could induce skeletal muscle atrophy.^37^ In our study, the anti-inflammatory effect of FER and DFER could contribute to the inhibition of skeletal muscle atrophy induced by obesity.

In addition to preventing muscle atrophy, our results demonstrated an improvement in glucose homeostasis within muscle tissue (see Figures 2 and 4). Vanillin, a compound naturally produced during curcumin degradation, has been shown to significantly reduce inflammation, enhance glucose uptake, and maintain muscle histology in diabetic rats when administered orally.^38^ Additionally, studies have highlighted the benefits of ferulic acid in promoting muscle glucose uptake^39^ and improving glucose tolerance by reducing pro-inflammatory responses and modulating gut microbiota, especially when combined with dietary fibers.^40^ Furthermore, curcumin and insulin exhibit a synergistic effect on glucose metabolism through the activation of the AMPK and PI3K/Akt pathways.^41^ Given their structural similarities, these studies may explain the beneficial effects of FER and DFER on glucose homeostasis.

Previous studies have suggested that gut microbial dysbiosis in obese subjects may contribute to higher levels of inflammation.^42^ In this study, HFD feeding resulted in a distinct gut microbial composition compared to the ND control group (Figure 6E,F). *Romboutsia illealis*, with high abundance in the HFD group, was found to increase in a colitis model, suggesting its potential correlation with inflammatory response.^43^ FER supplementation significantly reduced its growth, whereas DFER did not, possibly because DFER facilitated the growth of *Roseburia inulinivorans* DSM 16841, which is positively correlated with *R. illealis*. Members of the genus *Roseburia*, such as *Roseburia hominis*, are known butyrate producers.^44^*R. inulinivorans*, more abundant in the DFER than in the high-fat diet group, is recognized as a butyrate-producing bacterium beneficial for maintaining intestinal integrity.^45^ However, a lower abundance of *R. inulinivorans* DSM 16841 in the HFD group indicates that DFER supplementation could be responsible for its growth.

The gut microbial guild composed of *Blautia intestinalis*, *Dubosiella newyorkensis*, *Faecalicatena fissicatena*, *Waltera intestinalis*, *Clostridium viride*, and *Caproiciproducens galactitolivorans* was observed in both supplemented groups. Compared to single isolates, gut microbiome guilds could be more ecologically meaningful for explaining the role of gut microbiota in specific diseases or functions.^46^ Although the function of *B. intestinalis* has not been well studied, the genus *Blautia* has been identified as contributing to the maintenance of colonic mucus function through the secretion of short-chain fatty acids.^47^*D. newyorkensis* was previously found to modulate immune tolerance^48^ and exhibit an antiaging effect by increasing superoxide dismutase (SOD) levels in aged mice.^49^*C. viride*, also known as *Clostridium aminovalericum*, is capable of converting 5-aminovalerate to ammonia and various short-chain fatty acids (SCFAs), including acetate, propionate, and valerate.^50^ Lastly, *C. galactitolivorans* is a member of the *Caproiciproducens* genus that has the ability to produce lactate, caproate, and various SCFAs by consuming different sugars.^51^ Therefore, most members of this guild are known as potential SCFA producers. By directly or indirectly activating Free fatty acid receptor (FFAR)2 and FFAR3, SCFAs exhibit inhibitory effects on LPS-induced inflammatory responses,^52^ which may explain the ameliorative effects of FER and DFER on insulin resistance and muscle atrophy induced by HFD-related chronic inflammation.

In was previously reported that an increased abundance of gut microbes that utilize carbohydrates and promote carbohydrate metabolism may contribute to the development of insulin resistance.^53^ The increased abundance of Firmicutes in obese individuals may contribute to the activation of TLR2, TLR4, and CD14, which subsequently impairs the insulin signaling pathway.^54^ Additionally, various gut bacterial metabolites have been found to either exacerbate or alleviate insulin resistance.^55^ These metabolites may include indole and related compounds, phytochemical-derived metabolites, and conjugated lipids.^55^ These gut microbial metabolites could potentially improve sarcopenia by influencing energy metabolism, inflammation, and mitochondrial functionality.^56^ Additionally, extracellular vesicles derived from gut microbes may play crucial roles in immune responses, metabolism, and disease progression.^57^ Therefore, modulating gut microbiota holds strategic potential for preventing or treating obesity-related sarcopenia and insulin resistance. However, more comprehensive studies are needed to confirm this hypothesis. Our previous study suggested that FER and DFER supplementation could respectively facilitate the production of gut microbial acetic acid and butyric acid.^25^ It was found that SCFAs could potentially mitigate insulin resistance induced by LPS and TNF-α in differentiated C2C12 myotubes (Figure S6). Furthermore, correlation analysis (Figures S7–S9) revealed that fecal SCFAs were positively correlated with the muscle index and negatively correlated with fasting glucose levels, HOMA-IR, and pro-inflammatory cytokines in muscle tissue. A recent study demonstrated that SCFAs could recapitulate the obese skeletal muscle microenvironment by reducing the activation of NF-κB, thereby lowering the inflammatory response. Furthermore, higher concentrations of SCFAs could exert beneficial metabolic effects by stimulating glucose uptake in an obese environment.^58^ A clinical trial demonstrated that the gut microbiota and SCFAs are associated with skeletal muscle quality, but this association could be influenced by total body fat content.^59^ On the other hand, it was found that SCFAs could effectively suppress insulin-mediated fat accumulation by activating the short-chain fatty acid receptor GPR43.^60^ A systematic review and meta-analysis showed that postintervention levels of SCFAs have a beneficial effect on insulin sensitivity.^61^ These studies collectively suggest that gut microbiota-derived SCFAs play a crucial role in improving skeletal muscle quality, insulin sensitivity, and metabolic health, particularly in the context of obesity, by modulating inflammatory responses and activating specific receptors.

Our findings suggest that natural dietary supplements, such as curcuminoid degradants, could be a promising strategy for addressing obesity-related sarcopenia. These compounds exhibited a multitargeting effect, including antiobesity properties, promotion of muscle synthesis, prevention of muscle catabolism, anti-inflammatory actions, regulation of glucose homeostasis, and modulation of gut microbiota. While curcumin’s benefits are well-known, our results indicate that its thermal degradants, FER and DFER, may serve as effective alternative or complementary agents in dietary interventions aimed at mitigating obesity-related metabolic dysfunction.

In summary, our study demonstrated, for the first time, that supplementation of curcuminoid thermal degradants FER and DFER in a high-fat diet significantly improved various metabolic and inflammatory parameters. FER and DFER promoted muscle protein synthesis and reduced muscle protein degradation markers, suggesting a potential involvement of the PI3K/Akt/mTOR pathway, although further studies are required to confirm this mechanism. Additionally, they ameliorated insulin resistance and muscle atrophy associated with obesity-related chronic inflammation, potentially through modulation of gut microbiota. These findings suggest that FER and DFER could be effective dietary supplements for preventing obesity-related complications, but further research is needed to fully verify their mechanistic pathways and evaluate their long-term efficacy and safety.

## Data Availability

The data that support the findings of this study are available in the Supporting Information of this article.

## Supporting Information Available

Effect of different conditions on the differentiation of C2C12 Cells into myotubes, the effect of FER and DFER on glucose uptake ability of DEX-induced myotubes, the effect of FER and DFER on glucose uptake ability of DEX-induced myotubes, FER and DFER activate PI3K/Akt/mTOR in TNF-α-induced myotubes, both FER and DFER facilitate gut microbial guild formation in HFD-fed mice, SCFAs reverse abnormal glucose uptake induced by LPS or TNF-α in vitro, scatter plot showing the correlations between muscle index and fecal SCFA, scatter plot showing the correlations between insulin resistance indicators and fecal SCFA, and scatter plot showing the correlations between proinflammatory cytokines and fecal SCFA (PDF)

## Notes

The animal study was conducted following ethical guidelines and was approved by the Institutional Animal Care and Use Committee of the National Taiwan University with the approval number NTU-111-EL-00088.
