# Supplementary material for: Feruloylacetone and Its Analog Demethoxyferuloylacetone Mitigate Obesity-Related Muscle Atrophy and Insulin Resistance in Mice
Source: J Agric Food Chem. 2025 Jan 4;73(2):1231–43. doi: 10.1021/acs.jafc.4c07798 (PMC11741112; doi:10.1021/acs.jafc.4c07798)
Supplement: Supplementary file 1 — jf4c07798_si_001.pdf [file jf4c07798_si_001.pdf]

Supporting Material

Feruloylacetone and its Analog Demethoxyferuloylacetone Mitigate Obesity-Related Muscle Atrophy and Insulin Resistance in Mice

Yen-Chun Koh[†], Han-Wen Hsu[†], Pin-Yu Ho[†], Wei-Sheng Lin[†,‡], Kai-Yu Hsu[†], Anju Majeed[§], Chi-Tang Ho[||], Min-Hsiung Pan[†,#*]

[†] Institute of Food Sciences and Technology, National Taiwan University, 10617 Taipei, Taiwan

[‡] Department of Food Science, National Quemoy University, 89250 Quemoy, Taiwan

[§] Sami-Sabinsa Group Limited, Bengaluru, 560058 Karnataka, India

[||] Department of Food Science, Rutgers University, New Brunswick, 08901 New Jersey, USA

[#] Department of Medical Research, China Medical University Hospital, China Medical University, 40402 Taichung City, Taiwan

*** Please send all correspondence to:**
Dr. Min-Hsiung Pan
Institute of Food Science and Technology,
National Taiwan University,
No. 1, Section 4, Roosevelt Road, Taipei 10617, Taiwan.
Tel. no. +886-2-33664133
Fax. no. +886-2-33661771
E-mail: mhpan@ntu.edu.tw

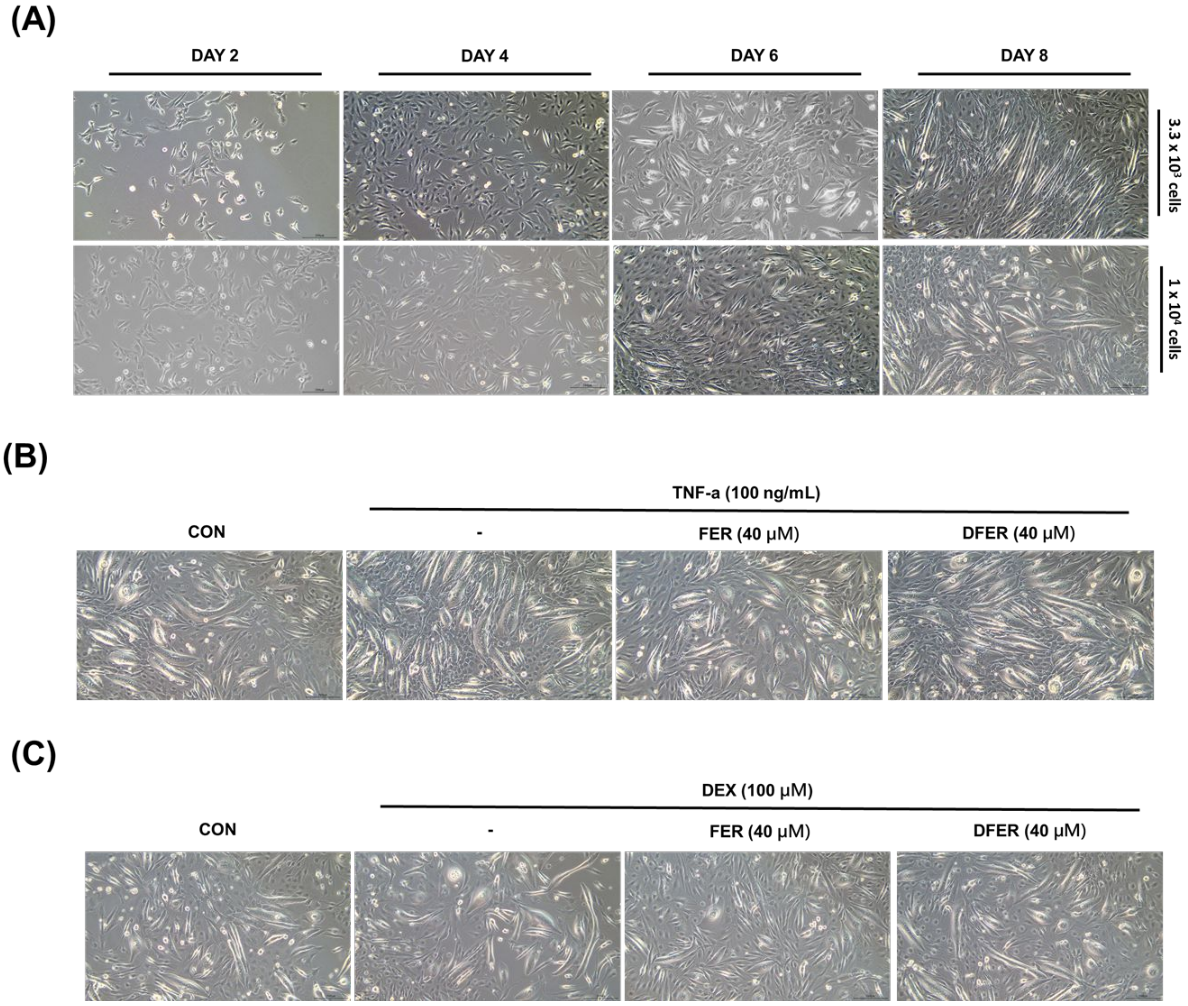

Figure S1. Effect of different conditions on the differentiation of C2C12 Cells into myotubes.

(A) C2C12 cells seeded at different densities were treated with 5% horse serum in DMEM medium for 8 days, the medium was changed every 2 days. Images of the differentiation process were recorded every 2 days. (B) Differentiated myotubes were treated with TNF-α (100 ng/mL) and either FER or DFER for 24 hours. (C) Differentiated myotubes were treated with DEX (100 μM) and either FER or DFER for 24 hours. Images were captured at 100x magnification.

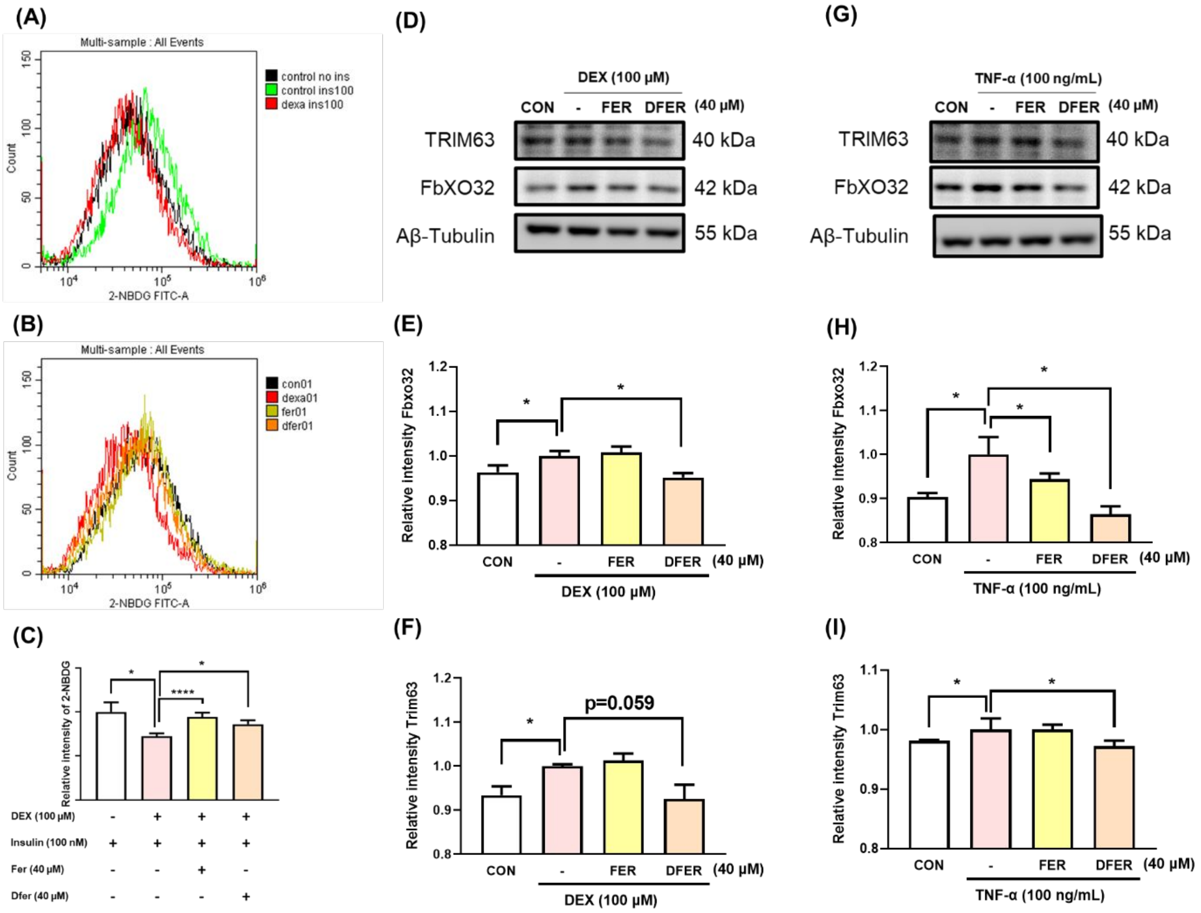

Figure S2. The effect of FER and DFER on glucose uptake ability of DEX-induced myotubes.

(A) Representative flow cytometry image showing 2NBDG uptake in DEX-induced myotubes. (B) Representative flow cytometry image showing 2NBDG uptake in DEX-induced myotubes after 24-hour intervention with FER and DFER. (C) Quantification of 2NBDG intensity in DEX-induced myotubes with and without intervention. (D) Representative western blot images showing the expression of FbXO32 and TRIM63 in DEX-induced myotubes after 24-hour intervention with FER and DFER. (E) Quantification of FbXO32 expression in DEX-induced myotubes after intervention with FER and DFER. (F) Quantification of TRIM63 expression in DEX-induced myotubes after intervention with FER and DFER. (G) Representative western blot images showing the expression of FbXO32 and TRIM63 in TNF-α-induced myotubes after 24-hour intervention with FER and DFER. (H) Quantification of FbXO32 expression in TNF-α-induced myotubes after intervention with FER and DFER. (I) Quantification of TRIM63 expression in TNF-α-induced myotubes after intervention with FER and

DFER. The symbols (*) indicates significant differences compared to the induced group, with *p*-values less than 0.05, determined by Student's t-test.

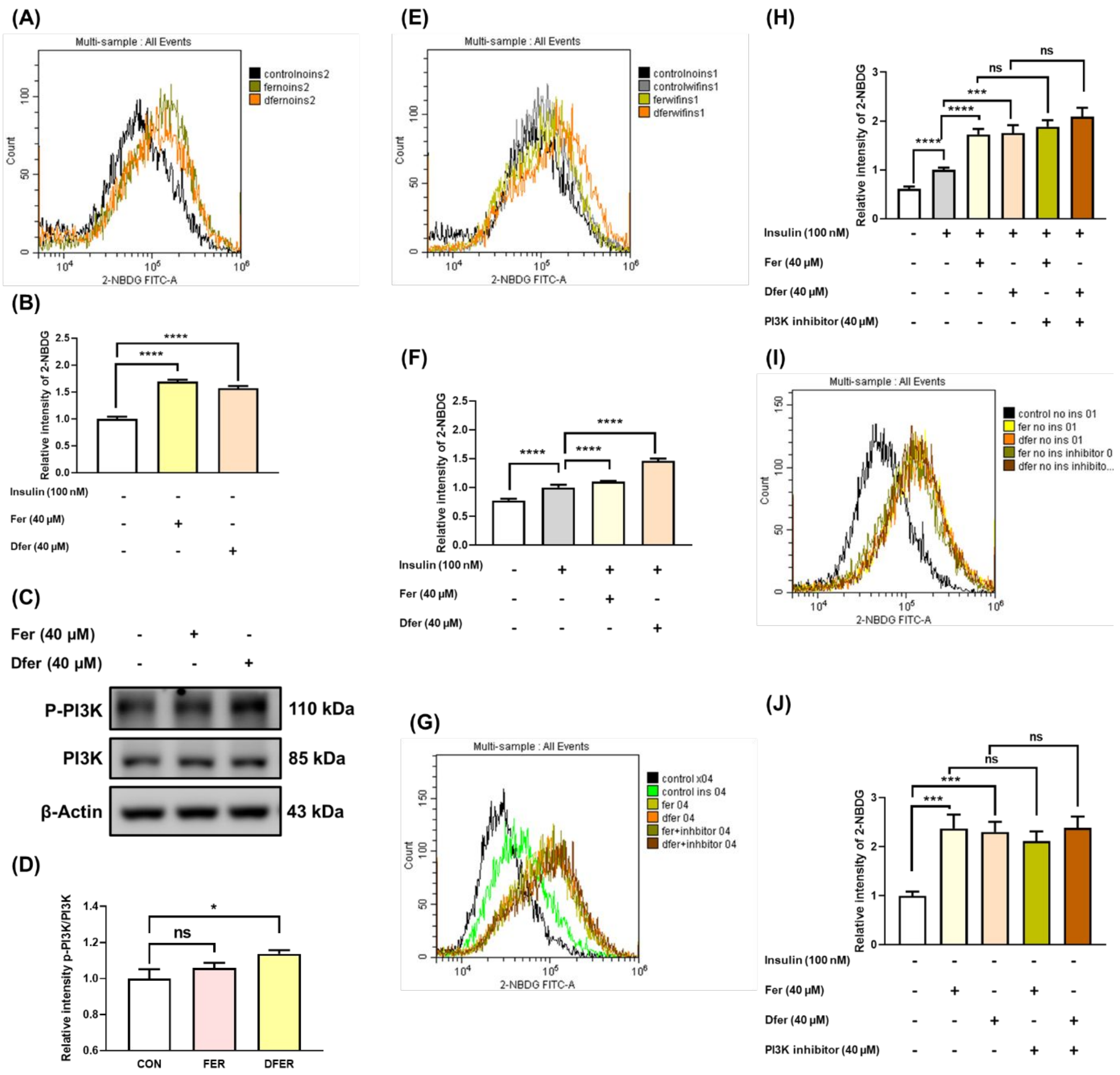

Figure S3. The effect of FER and DFER on glucose uptake ability of DEX-induced myotubes.

(A) Representative flow cytometry image and (B) quantification showing 2NBDG uptake without supplementation of insulin after 24-hour intervention with FER and DFER. (C) Representative western blot images and (D) quantification showing the expression of p-PI3K/PI3K after 24-hour intervention with FER and DFER. (E) Representative flow cytometry image and (F) quantification showing 2NBDG uptake with supplementation of insulin after 24-hour intervention with FER and DFER. (G) Representative flow cytometry image and (H) quantification showing 2NBDG uptake with supplementation of insulin after 24-hour intervention with FER, DFER and PI3K inhibitor. (I) Representative flow cytometry image and (J) quantification showing 2NBDG uptake without supplementation of insulin after 24-hour intervention with FER,

DFER and PI3K inhibitor. The symbols (*), (***) and (****) indicate significant differences compared to the induced group, with *p*-values less than 0.05, 0.005 and 0.001, respectively, determined by Student's t-test.

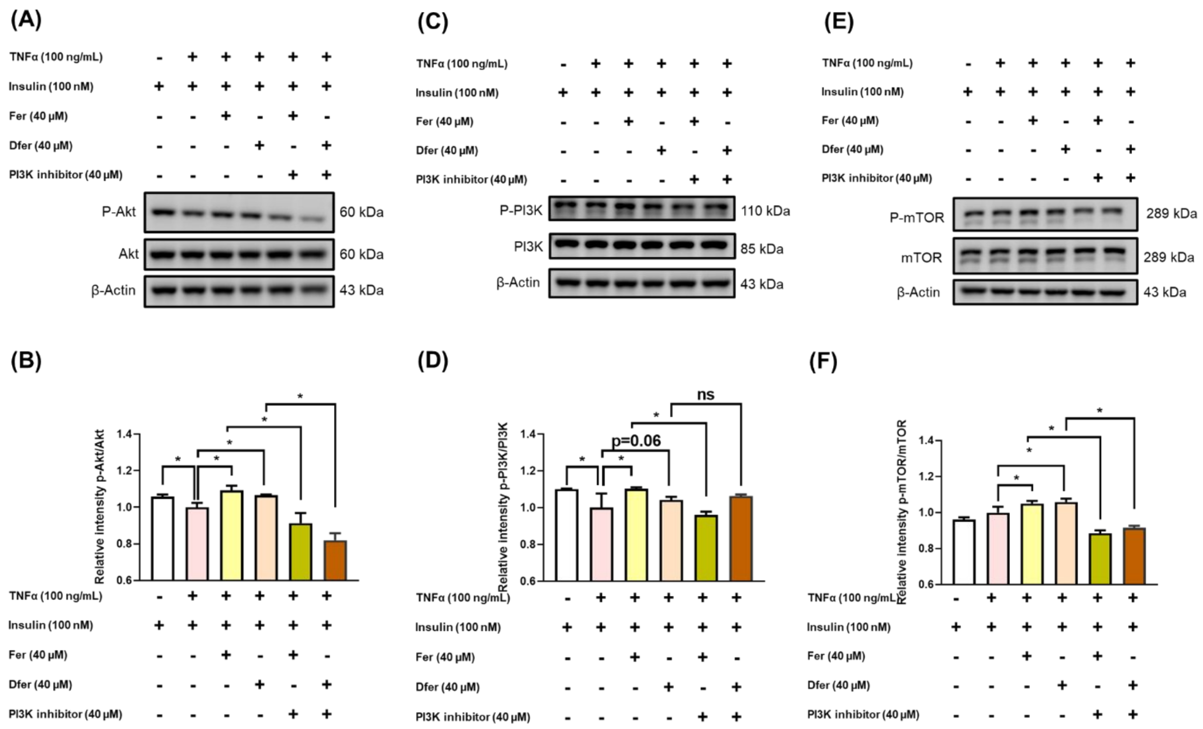

Figure S4. FER and DFER activate PI3K/Akt/mTOR in TNF-α-induced myotubes. (A) Representative western blot images showing the expression of p-Akt/AKT in TNF-α-induced myotubes after 6-hour intervention with FER and DFER. (B) Quantification of p-Akt/AKT expression in TNF-α-induced myotubes after intervention with FER and DFER. (C) Representative western blot images showing the expression of p-PI3K/PI3K in TNF-α-induced myotubes after 6-hour intervention with FER and DFER. (D) Quantification of p-PI3K/PI3K expression in TNF-α-induced myotubes after intervention with FER and DFER. (E) Representative western blot images showing the expression of p-mTOR/mTOR in TNF-α-induced myotubes after 6-hour intervention with FER and DFER. (F) Quantification of p-mTOR/mTOR expression in TNF-α-induced myotubes after intervention with FER and DFER. The symbols (*) indicates significant differences compared to the induced group, with *p*-values less than 0.05, determined by Student's t-test.

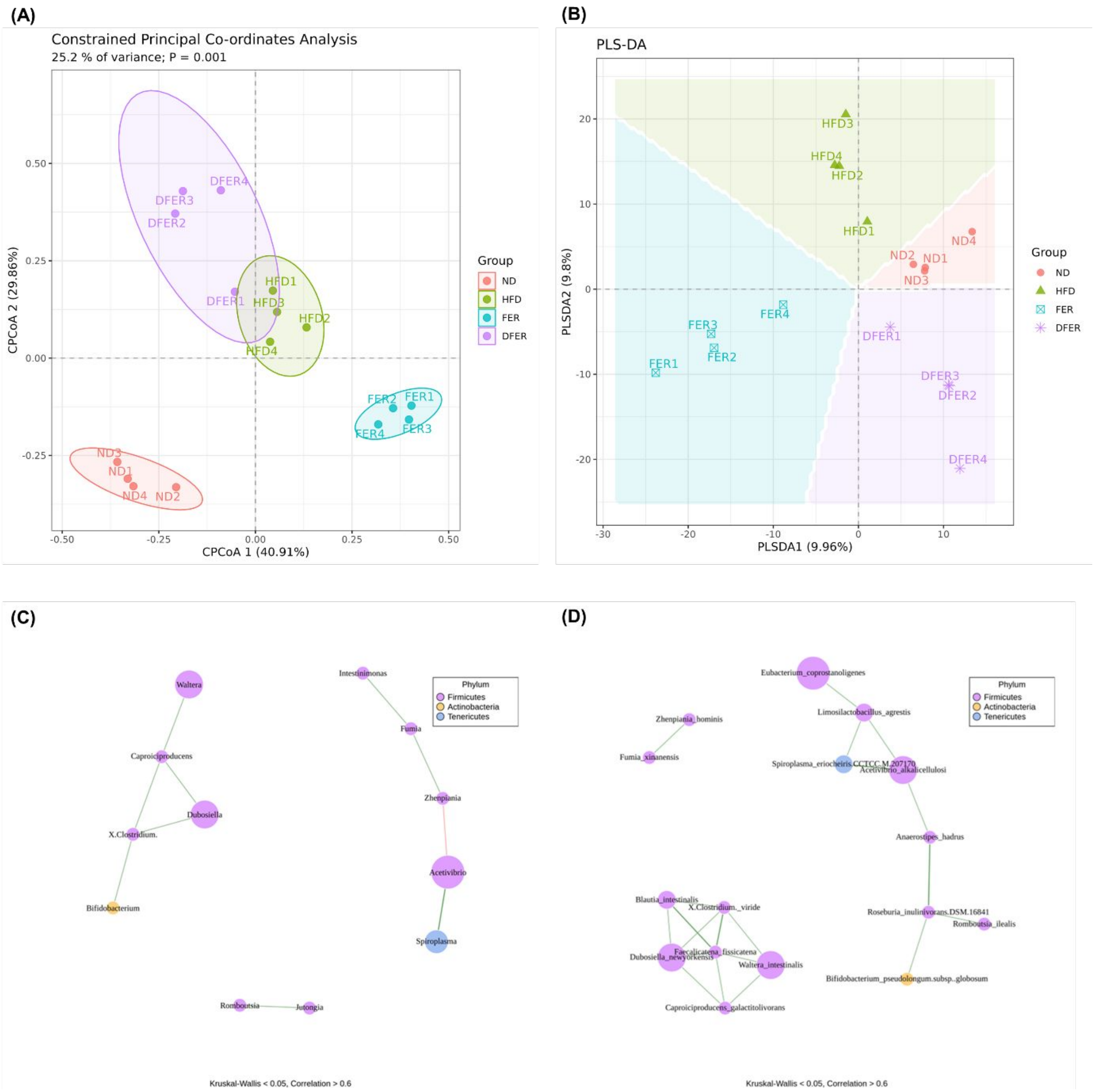

Figure S5. Both FER and DFER facilitate gut microbial guild formation in HFD-fed mice.

(A) CPCoA plot and (B) PLSDA plot. Correlation network at taxonomic rank of (C) genus and (D) species. Green lines between genera or species indicate positive correlations, while red lines indicate negative correlations. These correlations were found to be significant ($p < 0.05$) using the Kruskal-Wallis test.

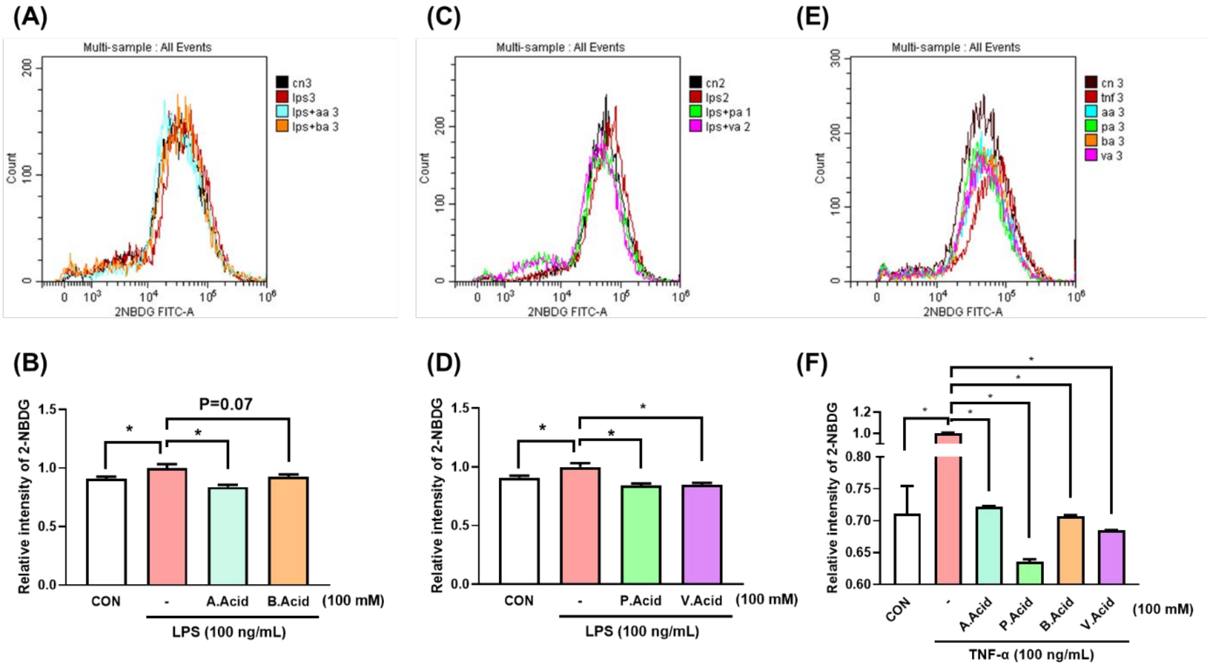

Figure S6. SCFAs reverse abnormal glucose uptake induced by LPS or TNF-α *in vitro*.

(A and C) Representative flow cytometry image and (B and D) quantification of 2NBDG uptake in LPS-induced cells treated with acetic acid, propionic acid, butyric acid or valeric acid. (E) Representative flow cytometry image and (F) quantification of 2NBDG uptake in TNF-α-induced cells treated with acetic acid, propionic acid, butyric acid or valeric acid. The symbols (*) indicates significant differences compared to the induced group, with *p*-values less than 0.05, determined by Student's t-test. A. Acid = acetic acid, P. Acid = propionic acid, B. Acid = Butyric acid and V. Acid = valeric acid.

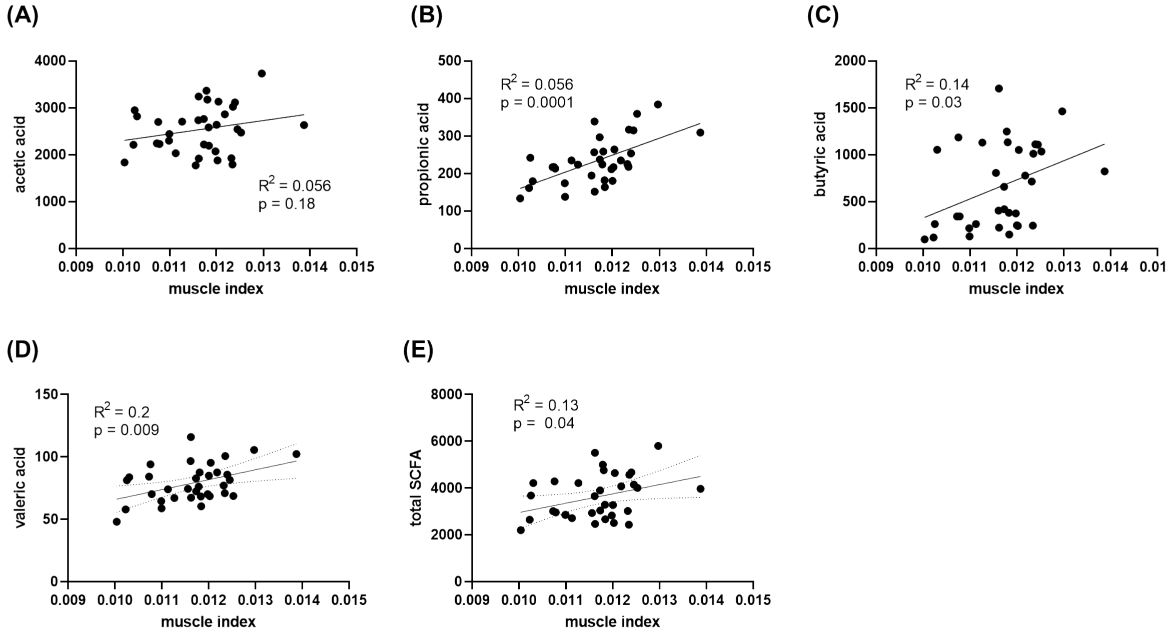

Figure S7. Scatter plot showing the correlations between muscle index and fecal SCFA.

(A) acetic acid, (B) propionic acid, (C) butyric acid, (D) valeric acid and (E) total SCFA. The correlation is considered significant (2-tailed) when $p < 0.05$.

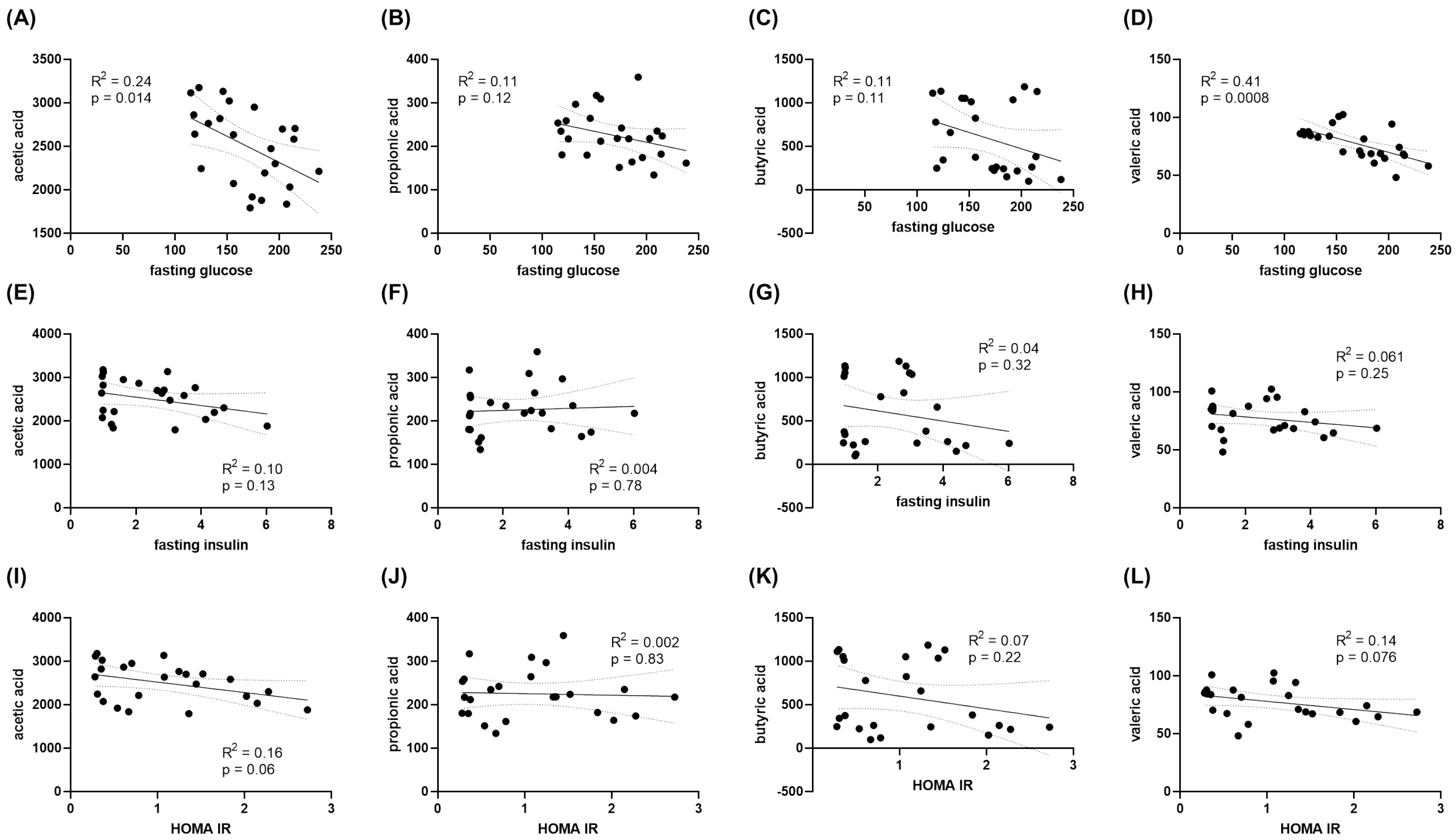

Figure S8. Scatter plot showing the correlations between insulin resistance indicators and fecal SCFA.

(A-D) Correlation between fasting glucose and fecal acetic acid, propionic acid, butyric acid, and valeric acid, respectively. (E-H) Correlation between fasting insulin and fecal acetic acid, propionic acid, butyric acid, and valeric acid, respectively. (I-L) Correlation between HOMA-IR and fecal acetic acid, propionic acid, butyric acid, and valeric acid, respectively. The correlation is considered significant (2-tailed) when $p < 0.05$.

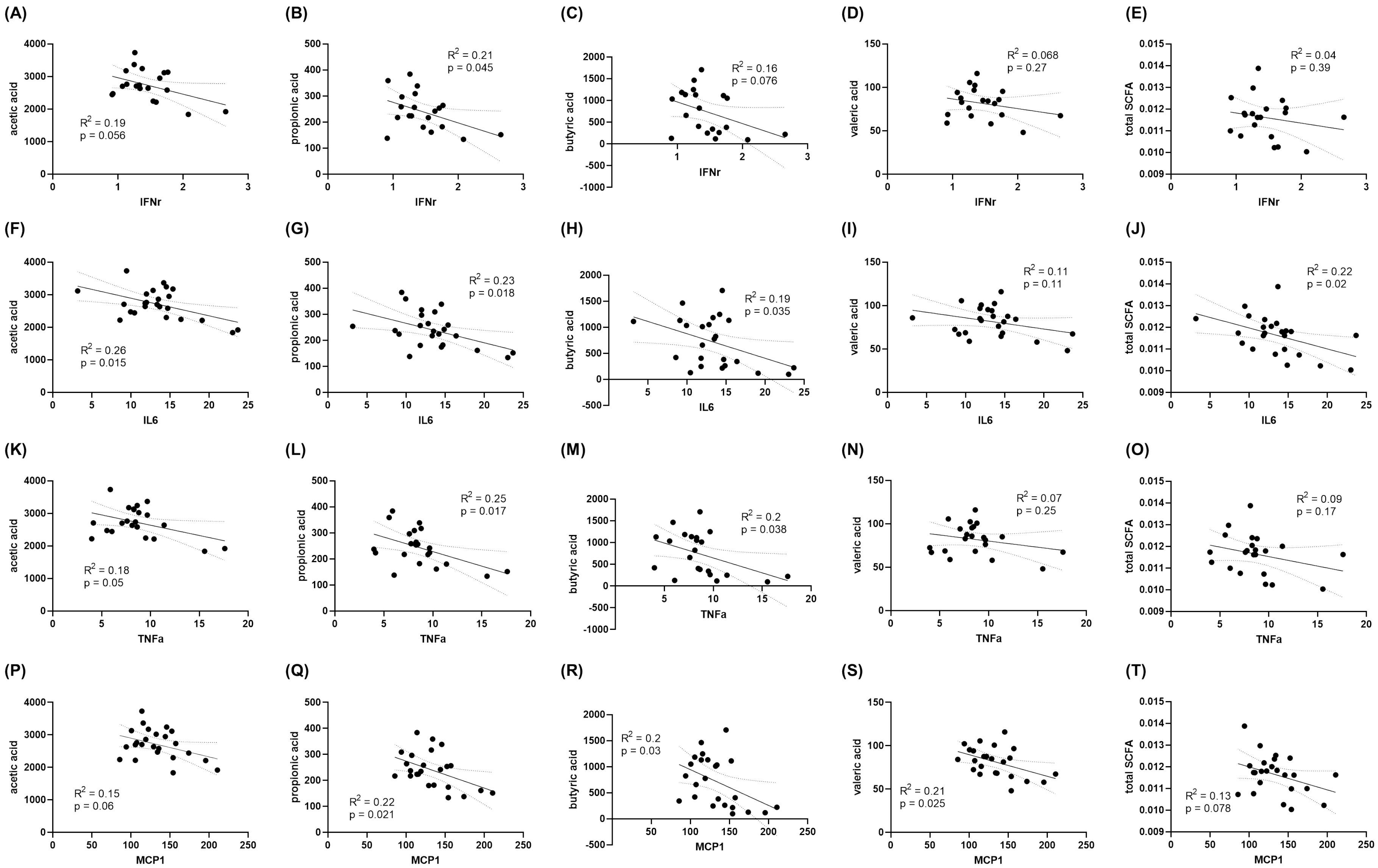
(A)
acetic acid
IFNr
R^2 = 0.19
p = 0.056
(B)
propionic acid
IFNr
R^2 = 0.21
p = 0.045
(C)
butyric acid
IFNr
R^2 = 0.16
p = 0.076
(D)
valeric acid
IFNr
R^2 = 0.068
p = 0.27
(E)
total SCFA
IFNr
R^2 = 0.04
p = 0.39
(F)
acetic acid
IL6
R^2 = 0.26
p = 0.015
(G)
propionic acid
IL6
R^2 = 0.23
p = 0.018
(H)
butyric acid
IL6
R^2 = 0.19
p = 0.035
(I)
valeric acid
IL6
R^2 = 0.11
p = 0.11
(J)
total SCFA
IL6
R^2 = 0.22
p = 0.02
(K)
acetic acid
TNFa
R^2 = 0.18
p = 0.05
(L)
propionic acid
TNFa
R^2 = 0.25
p = 0.017
(M)
butyric acid
TNFa
R^2 = 0.2
p = 0.038
(N)
valeric acid
TNFa
R^2 = 0.07
p = 0.25
(O)
total SCFA
TNFa
R^2 = 0.09
p = 0.17
(P)
acetic acid
MCP1
R^2 = 0.15
p = 0.06
(Q)
propionic acid
MCP1
R^2 = 0.22
p = 0.021
(R)
butyric acid
MCP1
R^2 = 0.2
p = 0.03
(S)
valeric acid
MCP1
R^2 = 0.21
p = 0.025
(T)
total SCFA
MCP1
R^2 = 0.13
p = 0.078

Figure S9. Scatter plot showing the correlations between proinflammatory cytokines and fecal SCFA.
(A-E) Correlation between muscle IFN-γ and fecal acetic acid, propionic acid, butyric acid, valeric acid, and total SCFA, respectively. (F-J) Correlation between muscle IL-6 and fecal acetic acid, propionic acid, butyric acid, valeric acid, and total SCFA, respectively. (K-O) Correlation between TNF-α and fecal acetic acid, propionic acid, butyric acid, and valeric acid, respectively. (P-T) Correlation between MCP-1 and fecal acetic acid, propionic acid, butyric acid, and valeric acid, respectively. The correlation is considered significant (2-tailed) when $p < 0.05$.
